# Novel Tumor-Targeted Self-Nanostructured and Compartmentalized Water-in-Oil-in-Water Polyurethane-Polyurea Nanocapsules for Cancer Theragnosis

**DOI:** 10.3390/pharmaceutics15010058

**Published:** 2022-12-24

**Authors:** Joaquín Bonelli, María Velasco-de Andrés, Neus Isidro, Cristina Bayó, Sergi Chumillas, Laura Carrillo-Serradell, Sergi Casadó-Llombart, Cheryl Mok, Daniel Benítez-Ribas, Francisco Lozano, Josep Rocas, Vicente Marchán

**Affiliations:** 1 Departament de Química Inorgànica i Orgànica, Secció de Química Orgànica, Institut de Biomedicina de la Universitat de Barcelona (IBUB), Universitat de Barcelona (UB), Martí i Franquès 1-11, E-08028 Barcelona, Spain; 2 Nanobiotechnological Polymers Division Ecopol Tech, S.L., El Foix Business Park, Indústria 7, L’Arboç del Penedès, E-43720 Tarragona, Spain; 3 Immunoreceptors del Sistema Innat i Adaptatiu, Institut d’Investigacions Biomèdiques August Pi i Sunyer (IDIBAPS), Rosselló 149-151, E-08036 Barcelona, Spain; 4 Servei d’Immunologia, Centre de Diagnòstic Biomèdic, Hospital Clínic de Barcelona, Villarroel 170, E-08036 Barcelona, Spain; 5 Departament de Biomedicina, Universitat de Barcelona (UB), Villarroel 170, E-08036 Barcelona, Spain

**Keywords:** nanoencapsulation, polyurethane-polyurea nanoparticles, tumor-targeting, drug delivery, bioimaging

## Abstract

Encapsulation of water-soluble bioactive compounds for enabling specific accumulation in tumor locations, while avoiding premature clearance and/or degradation in the bloodstream, is one of the main hallmarks in nanomedicine, especially that of NIR fluorescent probes for cancer theragnosis. The herein reported technology furnishes water-dispersible double-walled polyurethane-polyurea hybrid nanocapsules (NCs) loaded with indocyanine green (ICG-NCs), using a versatile and highly efficient one-pot and industrially scalable synthetic process based on the use of two different prepolymers to set up the NCs walls. Flow cytometry and confocal microscopy confirmed that both ICG-loaded NCs internalized in monocyte-derived dendritic cells (moDCs). The in vivo analysis of xenograft A375 mouse melanoma model revealed that amphoteric functionalization of NCs’ surface promotes the selective accumulation of ICG-NCs in tumor tissues, making them promising agents for a less-invasive theragnosis of cancer.

## 1. Introduction

Polymeric nanocapsules have emerged in recent years in nanomedicine as promising delivery systems of bioactive molecules [1]. Besides preventing degradation of the cargo molecules in the bloodstream, the multiple functionalization possibilities they offer through on-demand synthesis positions this class of polymeric constructions as powerful chemical tools in different medical fields [2], diagnosis and cancer treatment being two of the most studied [3].

Among polymeric nanocarriers, polyurethane-based nanocapsules (NCs) have demonstrated in recent years great biological performance and exceptional biocompatibility. By using the isocyanate-chemistry, unique structures exhibiting privileged properties can be designed and easily synthesized for applications in nanomedicine, as well as in other medical areas [4,5]. A broad spectrum of polyurethane-based nanomedical preparations have been described so far to address several biological challenges, such as increased dispersibility in aqueous media [6,7,8], selective cell or tissue drug vehiculization [9], promoted intracellular accumulation [10], and specific release under different external or internal stimuli [11,12].

Given the limitations that the enhanced permeability and retention (EPR) effect exhibits, mostly related to the variability of vessels extravasation in different types of solid tumors [13], a promising approach for targeting cancer consists of promoting the selective accumulation of drug nanocarriers into solid tumor acidic locations, which are generated by an aerobic glycolysis mechanism, known as Warburg’s effect. This process relays on the ability of cancer cells to generate energy, both under normoxic and hypoxic conditions, via a glycolytic pathway rather than through oxidative phosphorylation (OXPHOS), which is the common way that cells use to obtain energy from O_2_ in mitochondria. The lactate generated during aerobic glycolysis inside cancer cells is afterwards released to the external media, which contributes to pH depletion near to the solid tumor clustering [14]. This well-described phenomenon leads to pH values below 7, thereby opening the door to the design of nanocarriers that accumulate preferentially in acidic regions within the solid tumor microenvironment (TME). In a previous work, we have described a synthetic methodology for the one-pot production of completely crosslinked and long-circulating polyurethane-polyurea hybrid NCs, incorporating different liposoluble cargo molecules, which promote the selective accumulation in tumor locations by selective cationization of the nanocapsules’ surface when they reach the acidic TME. The amphoteric behavior of the nanocapsules was achieved through the incorporation of L-lysine and *N*-(3-dimethylaminopropyl)-*N,N*-diisopropanolamine on the polymeric wall [15,16]. Indeed, one of the tertiary amines in the latter compound has a p*K*_a_ value of approximately 7.2, which makes this group suitable to be selectively protonated under acidic pH conditions such as those found in the extracellular tumor microenvironment. However, the nanocapsules remain neutral at physiological pH (≈7.4) because the carboxylate groups of L-lysine residues incorporated within the polymeric wall are neutralized by the other protonated tertiary amine of *N*-(3-dimethylaminopropyl)-*N,N*-diisopropanolamine. In addition, release of the cargo can be triggered under reductive conditions (e.g., in cancer cells where the reduced form of glutathione peptide is highly overexpressed) owing to the incorporation of disulfide bonds in the polymer backbone [16]. Smart multi-stimuli-responsive nanocarriers are powerful tools in drug delivery since they can be designed to respond to chemical and/or biological stimuli [17,18,19,20]. Besides preventing the premature degradation and reticuloendothelial system (RES) evading [21] of the cargo, polyurethane-polyurea hybrid NCs have been described to increase the biological activity of a range of bioactive compounds (e.g., anticancer Ir(III)-based metallodrugs, immunosuppressors and coumarin-based photosensitizers for photodynamic therapy) by mediating intracellular vehiculization [22,23,24]. However, the nanomedical applications of polyurethane-based NCs are currently restricted to the encapsulation of liposoluble compounds, since stabilization in aqueous media takes advantage of the stratification of the polymeric wall in a hydrophobic-hydrophilic gradient (from the core to outwards of the nanocapsule, respectively).

The encapsulation and stabilization of hydrosoluble compounds in aqueous media embraced a lot of interest within the nanomedical community since it opens the door to the selective delivery both of small molecules and of therapeutically relevant biomolecules, such as proteins and oligonucleotides, among others. However, only a handful of nanostructures can actually fill this gap [25]. Liposomes have been positioned in recent years as a benchmark solution, owing to their high biocompatibility and synthesis feasibility. Unfortunately, liposomes exhibit several drawbacks to be considered as ideal nanocarriers, as they are poor crosslinked entities, which might lead to premature release of the cargo. The lack of reactive points liposomes difficults surface functionalization, which hampers using them in targeted therapy approaches [26].

Fluorophore-loaded nanocapsules, that can be externally monitorable (Bioimaging) or activatable (Phototherapy) with non-toxic and highly penetrating near infrared (NIR) light, have also gained broad interest recently for the development of novel non-invasive anticancer theragnostic devices [27,28,29]. However, only a limited number of examples of nanomedicines exploiting vehiculization of fluorescent probes in current medical use have been described so far. Hence, readdressing the biological target of FDA-approved organic fluorophores, such as Indocyanine Green (ICG, IC-Green™), Fluorescein (FLUORESCITE™), Methylene Blue (PROVAYBLUE™) or 5-aminolevulinic acid (Gliolan™; the only that has been approved in oncology for intraoperative visualization in the resection of glioma) [30] would expand considerably their medical applications for cancer theragnosis [31,32]. In this context, this study describes a groundbreaking one-pot and industrially scalable methodology to produce biocompatible, bioligand-free, and completely crosslinked Water-in-Oil-in-Water (WOW) double-walled polyurethane-polyurea hybrid redox-responsive nanocarriers for tumor-targeting. As far as we know, this is the first system that allows for the use of self-stratified polyurethane-based NCs for the encapsulation of hydrosoluble bioactive molecules.

As a proof of concept for encapsulation system validation, as well as for cancer targeting, the encapsulation of ICG has been addressed with the aim of producing new fluorescent probes for selective bioimaging [33]. The choice of ICG as a cargo molecule is especially interesting since this heptamethine cyanine derivative was one of the firsts NIR fluorescent probes approved by FDA for human clinical applications including cancer diagnosis. In addition, ICG-loaded nanocapsules can be easily monitorable by using a non-toxic/deep-penetrating multi-photon laser with chances for a streaming in vivo non-invasive tracking in animal models once injected. IVIS^®^ technology acknowledge the use of ICG, with a maximum of absorption centered at approximately 790 nm, for non-invasive longitudinal in vivo live imaging [34]. This is not the case of the other above-mentioned FDA-approved fluorescent probes that cannot be excited above 750 nm. Moreover, ICG can be used to destroy tumor cells and tissues by producing highly cytotoxic singlet oxygen as well as photothermal heat upon NIR irradiation, thereby combining diagnosis and therapy in a single agent [35]. Besides providing cancer targeting capacity, ICG encapsulation in stable and robust nanocapsules intends to avoid the well-described problems of this fluorophore, such as aggregation in aqueous media and premature degradation in plasmatic conditions [36], extending its absorption and fluorescence in time. In addition, encapsulation could be exploited to avoid rapid clearance of ICG by the liver as a consequence of non-specific binding to human serum albumin [35].

## 2. Materials and Methods

### 2.1. Synthetic Procedures

Indocyanine Green (ICG) was purchased from TCI Chemicals (Tokyo, Japan), whereas COUPY dye was synthesized following previously reported procedures [37,38,39], as described in the Supplementary Materials. The use of dry solvents is mandatory during the synthesis of the prepolymers to prevent degradation of isocyanate groups located at the polymeric chain ends by water.

#### 2.1.1. Synthesis of Redox-Responsive Amphiphilic Low HLB Prepolymer (P1)

2,2′-Dihydroxyethyl disulfide (DEDS) (2.86 g, 37.08 meq) and YMER N-120 (3.64 g, 7.01 meq) were added into a three-necked round-bottom flask equipped with mechanical stirring at room temperature (rt) and purged with N_2_. When the mixture was homogeneous, isophorone diisocyanate IPDI (8.83 g, 79.40 meq) was added into the reaction vessel under gentle mechanical stirring, checking the initial NCO stretching band intensity by FTIR. The polyaddition reaction was kept under these conditions until the NCO stretching band reach stabilization. At this point, dry acetone (18.2 mL) was added into the reaction mixture to fluidify the polymer. In parallel, 1,3-diamino-*N*-octadecylpropane (Genamin TAP 100D) (9.25 g, 56.72 meq) was dissolved in dry acetone (8.1 mL) into another 100 mL three-necked round-bottom flask, which had previously been purged with N_2_. The former reaction mixture was added dropwise onto the latter under 100 rpm mechanical stirring using a moon-shaped stirrer. The reaction was monitored by IR until the NCO stretching band intensity had completely disappeared.

#### 2.1.2. Synthesis of Redox Responsive Amphiphilic Cationic Prepolymer (P2)

2,2′-Dihydroxyethyl disulfide (DEDS) (901.0 mg, 11.68 meq), YMER N-120 (12.04 g, 23.18 meq) and *N*-(3-dimethylaminopropyl)-*N*,*N*´-diisopropanolamine (Jeffcat DPA) (981.3 mg, 8.99 meq) were added into a three-necked round-bottom flask equipped with mechanical stirring at room temperature (rt) and purged with N_2_. When the mixture was homogeneous, IPDI (8.14 g, 73.24 meq) was added into the reaction vessel under gentle mechanical stirring. The polyaddition reaction was kept under these conditions until the NCO stretching band intensity did not change, monitored by IR spectroscopy. At this point, dry THF (21 mL) was added into the reaction mixture to fluidify the polymer. In parallel, 1,3-diamino-*N*-octadecylpropane (Genamin TAP 100D) (5.99 g, 35.45 meq) was dissolved with dry THF (5.23 mL) into another 100 mL three-necked round-bottom flask, which had previously been purged with N_2_. The former reaction mixture was added dropwise onto the latter under 100 rpm mechanical stirring using a moon-shaped stirrer. The reaction was monitored by IR until the NCO stretching band intensity had completely disappeared.

#### 2.1.3. Synthesis of Dye-Loaded Cationic Redox Responsive Water-in-Oil-in-Water NCs (Cationic Dye-NCs)

The organic dye (ICG or COUPY) (5 mg), Milli-Q water (100 mg), diethylenetriamine (DETA) (4.5 mg, 0.13 meq) and prepolymer P1 (635.2 mg, 0.09 meq) were mixed in a 3 mL vial provided with magnetic stirring and protected from light. Then, 1.28 mL of cyclohexane were dropwise added to generate the water-in-oil emulsion. Afterwards, IPDI (40 mg, 0.36 meq) was used to crosslink the polymeric entity to furnish the first water-in-oil encapsulation (ENCAP 1), leaving most of NCO groups in the surface of NCs to be reacted in further steps.

To start the second encapsulation, the ENCAP 1 was added over a P2 solution (5.87 g, 0.61 meq) and it was properly mixed under magnetic stirring to consume the previous unreacted NCO groups. IPDI (225.0 mg, 2.02 meq) was then added into a three-neck round bottom flask, and provided with mechanical stirring, purged with N_2_, as well as protected from light. The mixture containing ENCAP 1 and P2 was then dropwise added over the IPDI at 100 rpm, checking the reappearance of NCO stretching band by FTIR. The resulting viscous organic phase was then emulsified at 450 rpm with cold Milli-Q water (12.0 g) and, after double-checking the presence of NCO stretching band by FTIR, a 10% (*w*/*w*) aqueous solution of DETA (36.12 mg, 1.05 meq) was finally added to generate completely crosslinked double-walled NCs from the nano micelles, consuming the NCO moieties. The stirring was then reduced to 100 rpm. Once the NCs were formed, THF, cyclohexane and acetone were removed from the reactor at 35 °C under vacuum and the dialysis purification was carried out using a molecular porous membrane tubing with a 12–14 kDa MWCO for 48 h against Milli-Q water.

#### 2.1.4. Synthesis of Dye-Loaded Amphoteric Redox Responsive Water-in-Oil-in-Water NCs (Amphoteric dye-NCs)

Dye-containing amphoteric NCs were prepared following the procedure described for cationic NCs (Section 2.1.3). Once the mixture containing ENCAP 1 and P2 had been dropwise added over the IPDI at 100 rpm, which resulted in the reappearance of the NCO stretching band by FTIR, an alkaline aqueous solution of L-lysine was prepared by dissolving 0.93 g of L-lysine in 11.37 g of Milli-Q water and adjusting pH to 11.0 with NaOH solutions of 3 M and 1 M (total l-lysine concentration 7.66% by wt). Then, 0.85 g of this solution (65.20 mg of L-lysine, 0.78 meq) were added at 350 rpm and the polyaddition reaction was checked immediately by FTIR. This viscous organic phase was then emulsified at 450 rpm with cold Milli-Q water (12.00 g) and, after double-checking the presence of NCO stretching band by FTIR, a 10% *w*/*w* aqueous solution of DETA (9.43 mg, 0.27 meq) was finally added to generate completely crosslinked double-walled NCs from the nano micelles, consuming the NCO moieties. The stirring was then reduced to 100 rpm. Once the NCs were formed, THF, cyclohexane and acetone were removed from the reactor at 35 °C under vacuum and the dialysis purification was carried out using a molecular porous membrane tubing with a 12–14 kDa MWCO for 48 h against Milli-Q water.

### 2.2. Biological Studies

#### 2.2.1. Monocyte-Derived Dendritic Cells (moDCs) Generation

Buffy coats from healthy patients were obtained from the *Banc de Sang i Teixits* (Barcelona, Spain). Peripheral blood mononuclear cells (PBMCs) were isolated by gradient centrifugation on ficoll lymphoprep^TM^ (STEMCELL technologies, Grenoble, France). PBMCs were suspended in 15 mL of X-VIVO medium (BioWhittaker, Lonza, Belgium) in t75 cm^2^ culture flasks and incubated for 2 h at 37 °C in a 5% CO_2_ atmosphere to promote monocyte adherence. Non-adherent cells were discarded and 12 mL of X-VIVO medium plus 2% human AB serum (HS), IL-4 (300 U/mL) and GM-CSF (450 U/mL) (Miltenyi Biotec, Madrid, Spain) were added to the flasks for further 72 h-incubation at 37 °C and in 5% CO_2_. Media was renewed on day 3 (12 mL X-VIVO plus HS, IL-4 and GM-CSF) and cells were recovered 72 h later (day 6), when fully derived into immature moDCs (i-moDCs) [40].

#### 2.2.2. Fluorescence Imaging by Confocal Microscopy

i-moDCs were generated as previously described and separated into 4 different conditions in 8 well µslide chambers (ibidi, Gräfelfing, Germany), at a concentration of 50.000 moDCs/800 µL X-VIVO-15 medium plus 2% HS, where nanocapsules were added at 2 µM of each cationic or amphoteric ICG-NCs. Cells were incubated with nanocapsules for 2 h at 37 °C and 5% CO_2_ to promote internalization and cell adherence. Multiphoton irradiation at 790 nm and detection centered at 810 nm conditions were set up for the internalization analysis.

For colocalization experiments, all conditions were stained with Lysotracker green and Hoechst as a nuclei counterstain, following the supplier’s instructions. After staining, wells were washed twice by carefully removing media without touching the well’s bottom and adding room temperature phosphate buffered saline (PBS) through the side. Finally, 800 µL of PBS was added to all wells and cells were observed under a Leica TCS SP5 laser scanning confocal microscope (Leica Microsystems Heidelberg GmbH, Mannheim, Germany) equipped with a DMI6000 inverted microscope, diode 405 nm, Argon Laser, HeNe 633 nm laser and 63× oil immersion objective lens (NA 1.4) and an incubation system with temperature, humidity and CO_2_ control was used. i-moDCs growing on 8 well micro slide ibidi chambers were labelled with Indocyanine Green and Lysotracker Green (Thermo Fisher Scientific, Waltham, MA, USA).

Lysotracker Green and ICG images were acquired sequentially using 488 and 633 laser lines, AOBS (Acoustic Optical Beam Splitter) as beam splitter and emission detection ranges 500–550 nm, 660–795 nm respectively and the confocal pinhole set at 1 Airy unit. Simultaneously, bright field transmitted light images were acquired. Images were acquired at 400 Hz in a 1024 × 1024 pixels format, and pixel size of 118 × 118 nm. Colocalization analysis was performed using JACOP plugin [41], a colocalization analysis tool from ImageJ (rsb.info.nih.gov/ij, accessed on 15 July 2021). Manders and Pearson coefficients were calculated for every set of images.

#### 2.2.3. Internalization Evaluation by Flow Cytometry

i-moDCs were cultured in 6 well plates in 3 mL X-VIVO 15 2% HS (0.5 × 10^6^ moDCs/mL) and incubated with different concentrations of either cationic COUPY-NCs, or amphoteric COUPY-NCs together with a blank (non-loaded Nanocapsules) in parallel at 37 °C and 4 °C for 6 and 24 h to observe whether internalization was conducted through active phagocytosis. Concentrations for cationic COUPY-NCs (experimental polymer value of 174.35 ± 0.26 mg/mL) were: (A) 0.619 µL/mL, (B) 1.55 µL/mL, (C) 3.1 µL/mL, (D) 6.19 µL/mL and (E) a blank of 3.29 µL/mL non-loaded NCs. Concentrations for amphoteric COUPY-NCs (experimental polymer value of 87 ± 5 mg/mL) were: (A) 1.24 µL/mL, (B) 3.105 µL/mL, (C) 6.21 µL/mL, (D) 12.42 µL/mL and (E) a blank of 1.61 µL/mL non-loaded NCs. After incubations, cells were detached and analyzed by flow cytometry (Attune NXT) to determine fluorescence on the green laser excitation (COUPY emission at 561 nm), with no other fluorochromes added.

#### 2.2.4. In Vivo Safety Assay of Amphoteric and Cationic Control NCs

All research procedures carried out in the user center were evaluated by the Ethics Committee for Animal Experimentation of the University of Barcelona and Generalitat de Catalunya (code 176/18). Healthy mice were administered with three different concentrations (30, 35 or 40 mg/mL of NCs; single intravenous injection of 160 μL) of cationic and amphoteric control NCs to confirm the lack of toxicity associated to the carrier. Hematological parameters in blood and biological parameters in plasma were evaluated after 7-days of single injection, in healthy B57BL/6 mice, and compared with those obtained from the control group.

#### 2.2.5. In Vivo Fluorescence Imaging Biodistribution in Subcutaneous Tumor Mice Models

Female NSG mice (9 weeks old, Charles River) were challenged by s.c. injection of A375 (1 × 10^6^) cells on the right flank with a 23-gauge needle. Amphoteric ICG-NCs and cationic ICG-NCs were administered i.v. at day 14 post-tumor challenge at 35 mg/mL of NCs (160 μL, 50 μM of ICG). PBS and ICG free were injected to control groups. Fluorescence was monitoring by in vivo imaging system (IVIS; Perkin Elmer, Waltham, MA, USA) using excitation/emission wavelengths of 780 nm/845 nm.

## 3. Results and Discussion

### 3.1. Design and Synthesis of WOW Double Walled Polyurethane-Polyurea Redox-Responsive Hybrid NCs

**Figure 1:**
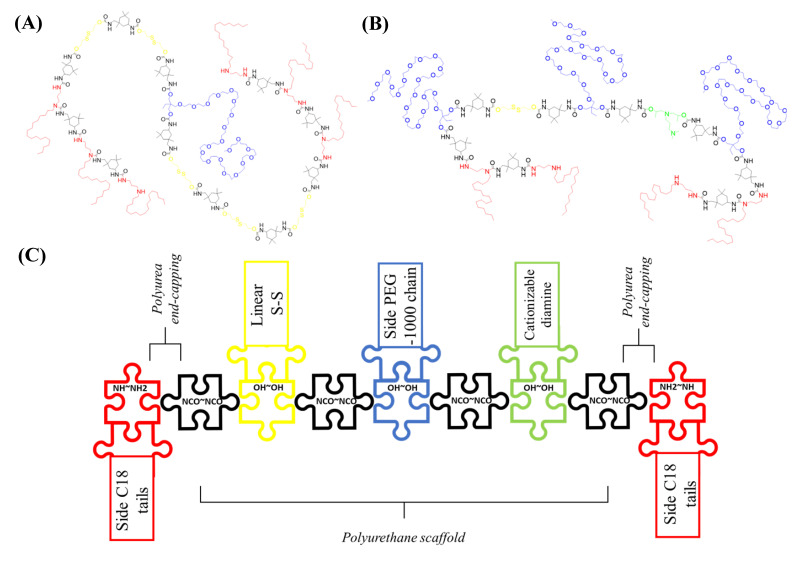
Chemical structure of polyurethane-polyurea hybrid prepolymers P1 (**A**) and P2 (**B**), and schematization of P2 synthesis (**C**), where the polyurethane backbone was initially designed to be afterwards end-capped with the amino groups from a hydrophobic diamine, furnishing the hybrid polyurethane-polyurea prepolymer.

**Figure 2:**
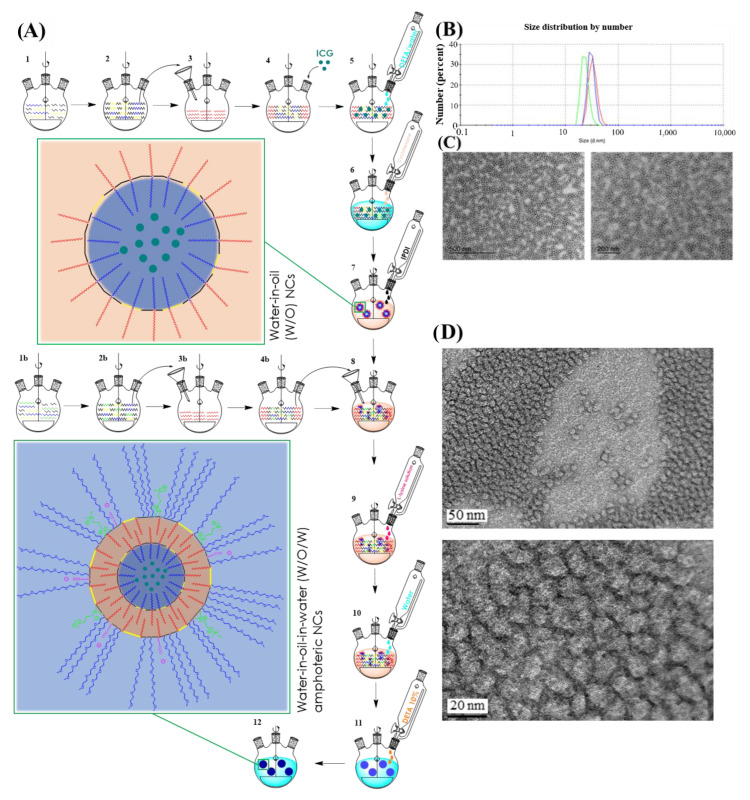
(**A**) Schematic synthesis of ICG-loaded amphoteric double-walled polyurethane nanocapsules. Steps 1 to 4 and 1b to 4b refer to the synthesis of both reactive polymers. Steps from 5–7 describe the synthesis of ICG-loaded W/O nanocapsules, while the following steps 8–12 exemplify the synthesis of final W/O/W ICG-loaded amphoteric nanocapsules. Magnifications of NCs structure after each crosslinking reactions are shown in step 7 and in step 12. Color codes are indicated in Figure 1C. (**B**) Particle size distribution by DLS of amphoteric ICG-loaded NCs in a triplicate analysis. (**C**) TEM micrographs of amphoteric ICG-loaded NCs and (**D**) the corresponding HR-TEM micrographs.

Inspired on the idea to reproduce liposomal arrangement, two different reactive prepolymers (P1 and P2) were first synthesized, with contrary properties in terms of Hydrophilic-Lipophilic Balance (HLB). Polyaddition reactions between a poly-isocyanate and polyalcohols or polyamines furnished the amino-capped hybrid polyurethane-polyurea hybrid structures. While both prepolymers include redox-responsive moieties to promote the polymer degradation under reductive conditions, P2 was also extra-functionalized with pH-responsive side-amines (Figure 1A,B). The creation of a first crosslinked water-in-oil nanocapsule was brought about using the self-emulsifying power of the low-HLB prepolymer P1, where a hydrosoluble molecule can be confined. This polymeric construction, which contains an increased ratio of lipophilic groups (C18-saturated side tails in red in Figure 1A) versus hydrophilic ones (PEGylated chains in blue in Figure 1A) generates a hydrophobicity gradient where the core-oriented PEGylated moieties settle the central aqueous phase surrounded by the first polymeric backbone, which exposes the lipophilic tails, stabilizing this water-core in an organic media (Figure 2A). The water-in-oil emulsion process is carried out as depicted in the first seven steps shown in Figure 2A. Immediately after polymer synthesis, an aqueous solution containing the hydrosoluble bioactive cargo molecules (Figure 2A; step 4) and a solution of 10% of DETA (Figure 2A; step 5) were added to the reaction flask and mixed with prepolymer P1. Dropwise addition of cyclohexane generates the W/O emulsion (Figure 2A; step 6), where the polymeric backbone of P1 is properly ordered at the interphase of the micelles. Then, the terminal secondary amino reactive functions contained in the polymer backbone, as well as the amino groups from the added DETA, were crosslinked by using a diisocyanate derivative (IPDI, Figure 2A; step 7) leading to the assembly, via the formation of stable urea bonds, of the internal water-in-oil nanocapsule construction (nanocapsule magnification in Figure 2A; step 7).

Next, the first crosslinked nanocapsule was dispersed in an organic media and mixed with the amino-capped high-HLB P2 prepolymer (Figure 2A; step 8). After the activation of P2 with an excess of diisocyanate (IPDI), which was confirmed by the appearance of the NCO stretching band according to FTIR analysis, the in situ reaction with the amino groups of L-lysine (Figure 2A; step 9) allowed to extend the hybrid polyurethane-polyurea chain, as well as to provide the system with pH-responsive capabilities owing to the incorporation of the α-carboxyl group of the basic amino acid. L-lysine addition step was only required for the synthesis of amphoteric nanocapsules, not for the formation of pure cationic ones. The system was then emulsified in water, leading to a “(water in oil) in water” system (Figure 2A; step 10), where an excess of reactive isocyanate was still present in the external interface of the emulsion. At this moment the hydrophobic tails were oriented to the core while PEGylated moieties stabilized the nanocapsule in aqueous dispersant phase. Upon addition of a polyamine (Figure 2A; step 11) and reaction with NCO groups, the crosslinking of the second polymeric backbone was achieved through the formation of urea bonds, producing the final WOW structure (Figure 2A; step 12 and corresponding magnification), where both original prepolymers P1 and P2 are defining the first and the second wall, respectively, without the need to use any external emulsifiers. Polyurethane-based prepolymers P1 and P2 were rationally designed to arrange their lateral groups in a water-in-oil interface, placing the polyurethane backbone in the appropriate location for an interfacial-promoted polyaddition with different reactants. This way to dispose stabilized reactive groups not only increases the crosslinking ability, but it would also facilitate the in situ functionalization of the nanocapsules [42]. As shown in Figure 2A, the “sandwich” attachment in the final product created by the interaction between hydrophobic tails of both polymers reminds the bilayer arrangement of lipidic and amphiphilic precursors in liposomal structures. However, it is important to note that in this case the confined water-based core remains completely isolated from the aqueous external medium by means of the two crosslinked polymers. As previously described for oil-in-water nanoencapsulation with polyurethane-polyurea-based systems [15,16,22,24], the introduction of disulfide bonds in the backbone of polymers P1 and P2 allows the degradation of the nanocarrier under reductive conditions. In addition, the incorporation of ionomeric groups on the polymeric wall provides the system with the required amphoteric behavior to facilitate accumulation in the acidic TME.

### 3.2. Synthesis and Characterization of ICG-Loaded NCs

Once set up the synthetic methodology for the preparation of WOW polyurethane-polyurea hybrid nanocapsules, the encapsulation of ICG was addressed. Water-dispersed ICG-loaded nanocapsules (ICG-NCs) were efficiently prepared following the abovementioned methodology with two different surface functionalities: cationic and amphoteric ICG-NCs. The fluorophore concentration in the nanocapsules was determined by UV-vis spectroscopy, showing good results for both encapsulation efficacy and dye-loading values (Table S4). The hydrodynamic diameter of ICG-loaded NCs was measured by Dynamic Light Scattering (DLS) (cationic; 55.22 ± 10.42 nm/amphoteric; 29.37 ± 5.25 nm) (Figure 2B) and their morphology and particle integrity were double-checked by Transmission Electron Microscopy (TEM) analysis (Figure 2C) and High-Resolution Transmission Electron Microscopy (HR-TEM) (Figure 2D). All TEM micrographs showed a roughly round shape and homogeneous particle size in good agreement with DLS measurements. It is worth noting that the size of double-walled water-in-oil-in-water polyurethane-polyurea hybrid nanocapsules was slightly higher compared with that of previously reported oil-in-water NCs [15,16,22,24], but still smaller than that of typical nanocarriers designed to facilitate tumor accumulation by the permeability and retention effect (EPR) [43]. This is an interesting attribute of the newly synthesized WOW NCs since smaller nanomedicines tend to exhibit higher tumor penetration [44].

In good agreement with the expected results, amphoteric ICG-NCs were found slightly smaller than the cationic ones, owing to the incorporation of the anionic group provided by L-lysine which acts as an ionomeric surfactant. Surface charge determination versus pH variations was conducted by ξ-potential measurements, showing that cationic nanocapsules slightly modified their surface charge on pH depletion, whereas a more significant change occurred in the case of amphoteric nanocapsules, which start from neutral charge at physiological pH and gradually become cationic after acidification to pH = 6.5 (Figure S14). The stability of amphoteric ICG-NCs was also investigated under different conditions and compared with that of the free form. As shown in Figure S17, the stability of the fluorescent probe was clearly improved upon nanoencapsulation, thus allowing to extend its imaging properties over the time.

### 3.3. In Vitro Cellular Uptake Evaluation of the NCs

**Figure 3:**
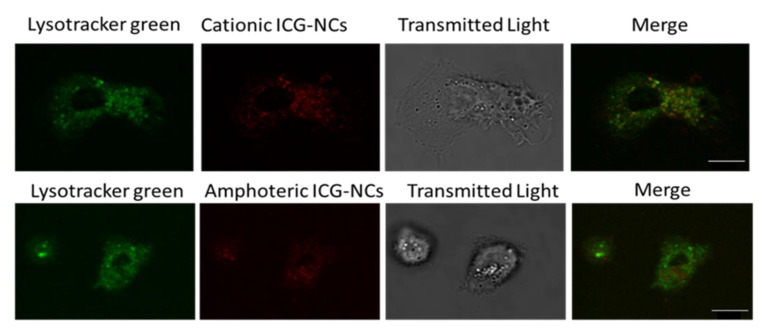
Cellular uptake by confocal microscopy of cationic and amphoteric ICG-loaded NCs in moDCs using multiphoton excitation. Both nanocapsules were incubated at 2 µM ICG concentration for 2 h at 37 °C. Scale bar: 10 μm.

A combination of Confocal Microscopy (Figures S18–S21) and Flow Cytometry (Figures S22–S25) studies were used to elucidate the mechanism of internalization for both cationic and amphoteric nanocapsules, as well as intracellular accumulation in Monocyte-derived Dendritic Cells (moDCs). Previously, cytotoxicity in moDCs was assessed with the viability marker 7-aminoactinomycin D (7-AAD), which stains exposed DNA—in this case from cellular death. One negative control was used in the experiment, consisting of immature DCs (iDCs). To our delight, cytotoxicity assays confirmed the absence of toxicity associated to ICG-loaded nanocapsules, since there was no significant difference between the associated cytotoxicity to NCs compared with untreated iDCs—in all the cases the associated cellular death was inferior to 30%. On one hand, cell internalization percentage was quantified by Flow Cytometry at 37 °C and 4 °C to determine the amount of nanocapsules crossing the cell membrane and to investigate the involvement of energy-dependent mechanisms. As ICG-loaded NCs require near infrared excitation, we decided to encapsulate a coumarin-based COUPY fluorophore with operability in the visible region of the electromagnetic spectrum to allow the use of a standard flow cytometer [37,38,39]. After 24 h incubation, both COUPY-loaded cationic and amphoteric nanocapsules reached high levels of internalization in moDCs, and energy-dependent pathways were demonstrated to be involved in the cellular uptake. As expected, cationic COUPY-loaded NCs were more rapidly internalized due to their enhanced penetrating properties. By contrast, the increasing internalization of amphoteric nanocapsules was more sustained in time compared with the cationic analogues since they deal with surface charge equilibria (Figure S23). On the other hand, the internalization of cationic and amphoteric ICG-loaded NCs in moDCs and in HeLa cells was investigated by confocal microscopy using multiphoton irradiation at 790 nm (detection at 810 nm). As shown in Figures S20 and S21, the fluorescence signal after incubation with both ICG-loaded NCs (2 μM, 30 min, 37 °C) was clearly observed inside the cells, mainly in the form of vesicles, indicating an excellent cell membrane permeability, even after shorter incubations times. Colocalization experiments with Lysotracker green (LTG) confirmed that most of the fluorescent vesicles were lysosomes (Figure 3).

### 3.4. In Vivo Fluorescence Imaging Biodistribution in Healthy Mice

Once demonstrated the good performance of ICG-loaded NCs upon multiphoton laser irradiation, we focused on validating the use of this methodology for cancer bioimaging in a complex live organism. For this purpose, a range of multiphoton in vivo live imaging experiments in different mice models using IVIS™ technology was designed.

In a zero stage of in vivo experimentation, the toxicity associated to the nanocarrier was first evaluated by single intravenous injection (tail vein) at three different concentrations in healthy mice for the subsequent evaluation, after one week, of the hematological parameters in blood and the biological parameters in plasma. Once demonstrated the absence of any side-toxicity associated to the nanocapsules since no differences between control and treated mice groups were found in terms of hematocrit analysis and body weight variation (Tables S5 and S6), we focused on investigating the biodistribution of amphoteric and cationic ICG-loaded NCs in mice models (Figures S26–S30). First, C57BL/6 mice received a testing concentration of cationic ICG-NCs in order to ensure the imaging viability. However, due to the dark hair color this mouse strain has, we were unable to properly detect fluorescent signal by IVIS and, consequently, we decided to euthanize mice to extract organs for ICG-loaded NC detection. Spleen, kidney and, specially, liver showed quite high accumulation (Figure S27). Based on these results, mouse strain selection was then shifted to a BALB/c albino strain to facilitate the in vivo ICG-mediated monitorization. Cationic and amphoteric ICG-loaded NCs were intravenously injected in a healthy BALB/c mouse, checking their biodistribution profile against free ICG for 48 h. All the mice (*n* = 4 and 2 controls per group) received the same concentration of ICG, in either free or the nanoencapsulated form. A clear improvement in the biodistribution performance of the ICG-loaded NCs was observed compared with the free fluorophore. Indeed, the fluorescence signal of the free ICG form was turned off before 24 h after injection (Figure S28), presumably because of a rapid clearance from mice body and/or degradation of the fluorescent probe. By contrast, while ICG-NCs were monitorable for more than 48 h-after intravenous administration, being heterogeneously distributed along the animal’s body. These observations confirm that polyurethane-polyurea hybrid nanocapsules increase the circulating time of ICG, preventing degradation, opsonization and/or RES-removal (Figures S29 and S30).

### 3.5. In Vivo Fluorescence Imaging Biodistribution in Subcutaneous Tumor Mice Model

**Figure 4:**
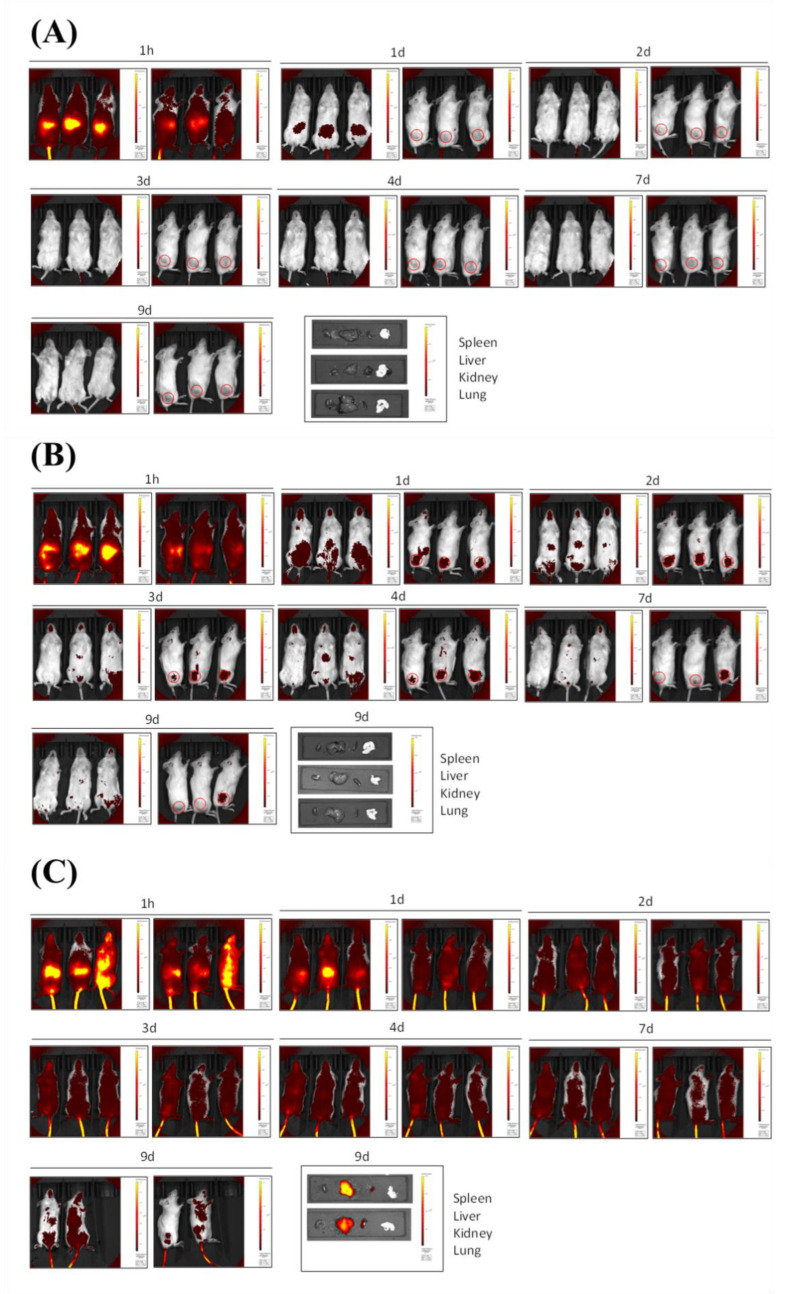
In vivo fluorescence imaging biodistribution in subcutaneous tumor mice models. Nine-days biodistribution pattern of (**A**) A375 challenged NSG mice treated with a 40 µM solution of ICG (*n* = 3/group), (**B**) A375 challenged NSG mice treated with amphoteric ICG-NCs (*n* = 3/group) at 40 µM ICG, and (**C**) A375 challenged NSG mice treated with cationic ICG-NCs (*n* = 3/group) at 40 µM ICG. Locations for tumor cells injections have been indicated with a red circle.

Finally, both ICG-loaded NCs were tested for selective accumulation in tumor locations in a melanoma xenograft model, implanting A375 cells in immunosuppressed NSG mice. This human tumor model was subcutaneously implanted on the right flank of the animals (red dashed circles tag tumor cells locations in Figure S31 and Figure 4), and it was allowed to grow for 14-days before ICG free or ICG-loaded NCs administration. Remarkably, for the same dose via intravenous administration, the ICG free signal disappeared on the second visualization day, 48 h after injection (Figure 4A), which could be attributed both to a rapid excretion from the body and to the degradation of the dye promoted by circulating biological agents. By contrast, the fluorescence signal from the dye in both amphoteric (Figure 4B) and cationic (Figure 4C) ICG-loaded NCs was clearly trackable for more than 7 days after injection, confirming the integrity of the nanocarrier in the bloodstream (*n* = 3 per group; control group is shown on Figure S31A). The lack of specificity for tumor accumulation of cationic ICG-NCs makes them more long-lasting than the amphoteric ones, which suggests that the latter are gradually degraded once accumulated in tumor cells located in mice right flank owing to the overexpression of glutathione.

In addition to these results, it is worth noting that amphoteric ICG-NCs operate as realistic tumor tag probes, clearly promoting the accumulation of ICG onto tumor locations (Figure 4B). By contrast, cationic ICG-NCs show higher signaling but decreased selectivity for tumor tissues (Figure 4C). The evaluation of the amount of fluorescence signal over liver, kidneys, spleen and lungs supported these conclusions since the dye was absent in the case of amphoteric ICG-NCs group 9-days after injection, whereas a larger amount of fluorescence signal was still found in excretion organs of the cationic ICG-NCs-administered group. In the case of well-boding amphoteric functionalized ones, accumulation in excretion organs, as a significant factor which could lead to side-toxicity inconveniences in the future, was not detectable at all.

Overall, these results are in good agreement with those previously obtained for liposoluble-drug loaded amphoteric polyurethane-polyurea hybrid NCs, where tumor accumulation was confirmed by post-euthanized fluorescence analysis of organs/tissues [16], unmasking the importance of the amphoteric surface functionalization of polymeric nanocapsules as a promising way to target solid tumor microenvironment.

## 4. Conclusions

In summary, we have developed and validated in a biologically relevant tumor xenograft model, a novel, versatile and highly efficient one-pot and industrially scalable synthetic process for the preparation of redox-responsive double-walled polyurethane-based nanocapsules as selective nanocarriers of water-soluble bioactive compounds. As a proof of concept, the FDA-approved fluorescent probe ICG was incorporated in the aqueous core of the nanocapsules, with both cationic and amphoteric surface properties, confirming their exceptional properties for bioimaging as well as for tumor targeted delivery. According to in vivo biodistribution studies in subcutaneous tumor-bearing mice models, amphoteric nanocapsules were found much more effective than the cationic ones since they were designed to selectively accumulate in the acidic tumor microenvironment. The accumulation in acidic tumoral zones can be attributed to the presence of an excess of lactic acid in peripheral tumor cell’s locations derived from Warburg’s effect. By contrast, cationic nanocapsules did not show any specific in vivo biodistribution towards tumor cell’s location. Furthermore, polyurethane-based control nanocapsules have been verified as non-toxic at all, both in vitro and in vivo, which position them as a powerful tool for tumor resection by fluorescence-guided surgery. Work is in progress in our laboratories to explore the use of amphoteric redox responsive water-in-oil-in-water nanocapsules as drug delivery systems of hydrophilic anticancer drugs with the aim of exploring clinical applications.

## 5. Patent

The encapsulation technology described in this article is protected by patent PCT/EP2022/058801, entitled “Nanotechnological platform based on polyurethane/polyurea chemistry to furnish water-oil-water multi walled and functionalizable nanocapsules and their preparation process” from Bonelli, J, and Rocas, J., granted to Ecopol Tech S. L.

## Data Availability

Data are contained within the article and Supplementary Materials.

## Supplementary Materials

The supporting information can be downloaded at: https://www.mdpi.com/article/10.3390/pharmaceutics15010058/s1.
