# Supplementary material for: Novel Tumor-Targeted Self-Nanostructured and Compartmentalized Water-in-Oil-in-Water Polyurethane-Polyurea Nanocapsules for Cancer Theragnosis
Source: Pharmaceutics. 2022 Dec 24;15(1):58. doi: 10.3390/pharmaceutics15010058 (PMC9862617; doi:10.3390/pharmaceutics15010058)
Supplement: Supplementary file 1 [file pharmaceutics-15-00058-s001.zip › pharmaceutics-2040551-supplementary.pdf]

SUPPLEMENTARY MATERIALS

Novel Tumor-Targeted Self-Nanostructured and Compartmentalized Water-in-Oil-in-Water Polyurethane-Polyurea Nanocapsules for Cancer Theragnosis

Joaquín Bonelli [1,2], María Velasco-de Andrés [3], Neus Isidro [2], Cristina Bayó [4], Sergi Chumillas [1], Laura Carrillo-Serradell [3], Sergi Casadó-Llombart [3], Cheryl Mok [4], Daniel Benítez-Ribas [4], Francisco Lozano [3,4,5], Josep Rocas [2] and Vicente Marchán [1,*]

[1] Departament de Química Inorgànica i Orgànica, Secció de Química Orgànica, Institut de Biomedicina de la Universitat de Barcelona (IBUB), Universitat de Barcelona (UB), Martí i Franquès 1-11, E-08028 Barcelona, Spain
[2] Nanobiotechnological Polymers Division Ecopol Tech, S.L., El Foix Business Park, Indústria 7, L'Arboç del Penedès, E-43720 Tarragona, Spain
[3] Immunoreceptors del Sistema Innat i Adaptatiu, Institut d'Investigacions Biomèdiques August Pi i Sunyer (IDIBAPS), Rosselló 149-151, E-08036 Barcelona, Spain
[4] Servei d'Immunologia, Centre de Diagnòstic Biomèdic, Hospital Clínic de Barcelona, Villarroel 170, E-08036 Barcelona, Spain
[5] Departament de Biomedicina, Universitat de Barcelona (UB), Villarroel 170, E-08036 Barcelona, Spain
* Correspondence: vmarchan@ub.edu

Table of contents

1. Materials

1.1. Building blocks and crosslinkers S4

1.2. Encapsulated molecules S4

1.3. Solvents and auxiliary solutions S4

1.4. Biological agents, mediums and supplements S4

2.- Analytical techniques

2.1. Infrared spectroscopy (IR) S6

2.2. pH measurements S6

2.3. Dynamic light scattering (DLS) S6

2.4. Zeta potential (Z-pot) S6

2.5. Dialysis purification S6

2.6. Determination of cargo loading by UV-vis spectroscopy S6

2.7. Solids concentration S7

2.8. Transmission electron microscopy (TEM) S7

2.9. High-resolution transmission electron microscopy (HR-TEM) S7

3.- Synthetic procedures.

3.1. Synthesis of redox-responsive amphiphilic low HLB prepolymer (P1) S8

3.2. Synthesis of redox responsive amphiphilic cationic prepolymer (P2) S9

3.3. Synthesis of dye-loaded cationic redox responsive water-in-oil-in-water NCs (cationic dye-NCs) S10

3.4. Synthesis of dye-loaded amphoteric redox responsive water-in-oil-in-water NCs (amphoteric dye-NCs) S11

3.5. Synthesis of non-loaded cationic and amphoteric redox responsive water-in-oil-in-water NCs (cationic and amphoteric control NCs) S11

3.6. Comparison of the structure of cationic and amphoteric ICG-loaded NCs S12

4.- Characterization of polymer **P1** and NCs

4.1. Infrared Spectroscopy S13

4.2. Average size of NCs by Dynamic Light Scattering S16

4.3. ζ -potential of non-loaded NCs S20

4.4. ICG and COUPY payload determination in NCs S21

4.5. Transmission electron microscopy (TEM) S22
4.6. Stability of amphoteric ICG-loaded NCs under different conditions S23

5.- Biological studies
5.1. Internalization evaluation of ICG-loaded NCs in human monocyte derived dendritic cells (moDCs) by confocal microscopy S24
5.2. *In vitro* Internalization studies in human monocyte derived immature DCs by Flow Cytometry S27
5.3. *In vivo* safety assay of amphoteric and cationic control NCs S30
5.4. Biodistribution of amphoteric and cationic ICG-loaded NCs in mice models
5.4.1 *In vivo* fluorescence imaging biodistribution in healthy mice.
5.4.1.1 Intravenous administration in C57BL/6 mice. S31
5.4.1.2 Comparison on excretion organs NCs signal between different administrations in C57BL/6 mice model S32
5.4.1.3 Intravenous administration biodistribution comparison between ICG, amphoteric ICG-NCs and cationic ICG-NCs at 1 h, 24 h and 48 h in healthy BALB/C mice model S33
5.4.2 *In vivo* fluorescence imaging biodistribution in subcutaneous tumor mice models S36

1. Materials

1.1. Building blocks and crosslinkers

Isophorone diisocyanate IPDI was purchased from Quimidroga (Barcelona, Spain), YMER N-120 was kindly supplied by Perstorp (Malmö, Sweden) and Genamin TAP 100D was provided by Clariant (Barcelona, Spain). Jeffcat DPA, DEDS (2-hydroxyethyl disulfide), DETA (diethylenetriamine) and L-lysine hydrochloride were purchased from Sigma Aldrich (St Louis, USA).

1.2. Encapsulated molecules

Indocyanine Green (ICG) was purchased from TCI Chemicals (Tokyo, Japan) whereas COUPY dye was synthesized as indicated in section 1.2.1.

1.2.1. Synthesis of 3-(4-(cyano(7-(*N,N*-diethylamino)-4-methyl-2H-chromen-2-ylidene)methyl)pyridin-1-ium-1-yl)propane-1-sulfonate (COUPY dye)

To a solution of 105 mg (0.32 mmol) of 2-(cyano(4-pyridine)methylene)-7-(*N,N*-diethylamino)-4-methyl-coumarin in ACN (30 mL), 1,3-propane-sultone (1.12 mL, 12.67 mmol) was added under an Ar atmosphere and the resulting mixture was stirred overnight at 90 ºC and protected from light. After evaporation to dryness and purification by column chromatography (silica gel, 0-20% MeOH in DCM), 83 mg of a red solid were obtained (yield: 58%). Characterization: TLC: Rf (DCM/MeOH 9:1) 0.3; ^{1}H NMR (DMSO-d_6, 400 MHz): δ (ppm) 8.69 (2H, d, J = 7.2 Hz), 8.22 (2H, d, J = 7.2 Hz), 7.75 (1H, d, J = 9.2 Hz), 7.01 (1H, s), 6.96 (1H, dd, J = 6.8 Hz), 6.91 (1H, d, J = 6.8 Hz), 4.55 (2H, t, J = 7.2 Hz), 3.55 (4H, q, J = 7.2 Hz), 2,51 (3H, s), 2.43 (4H, t, J = 7.2 Hz), 2.18 (2H, m), 1.18 (6H, t, J= 6.8 Hz); LRMS (ESI): *m/z* 454.11 calc. for $[C_{24}H_{27}N_3O_4S+H]^+$ = 454.18.

1.3. Solvents and auxiliary solutions

Milli-Q water was obtained from a Merck Millipore purification system (Madrid, Spain) and PBS, HCl 37% and NaOH in pellets were purchased from Merck (Madrid, Spain). Cyclohexane was purchased from Panreac AppliChem (Barcelona, Spain).

1.4. Biological agents, mediums, and supplements

Dulbecco's Modification of Eagle's Medium (DMEM) was purchased from Corning (Arizona, USA) and DMEM with low glucose, pyruvate and without glutamine and Phenol Red were purchased from Thermo Fischer Scientific (Massachusetts, USA), as well as Fetal Bovine Serum (FBS), Dulbecco′s Phosphate Buffered Saline (DPBS), Horse serum, Hoescht 33342 solution, Lysotracker Green (LGT) and Mytotracker Green (MTG). L-glutamine solution (200 mM), F12 (HAM) media and 100 U mL^{-1}

penicillin/100 μg mL^{-1} streptomycin were acquired from Biological Industries (Cromwell, USA). Hepes buffer (1M) was purchased from Lonza (Basel, Switzerland), as well as X-VIVO15 medium. Insulin, Hydrocortisone, Human Serum AB (HS) and Epidermal Growth Factor (EGF) were acquired from Sigma-Aldrich (St Louis, USA). Choleric toxin was purchased from Calbiochem (San Diego, CA, USA). The cytokines IL-4 and Granulocyte-Monocyte-Colony Stimulation Factor (GM-CSF) were purchased from Miltenyi Biotec (Bergisch Gladbach, North Rhine-Westphalia, Germany).

2. Analytical techniques

2.1. Infrared spectroscopy (IR)

IR spectra were performed in a Smart ATR (Nicolet iS10, Thermo Scientific, Raleigh, USA) using a transmittance mode (16 scans) and OMNIC software. For the monitoring of solvent-based samples, one drop was deposited onto the diamond crystal and the solvent was left to dry by evaporation. IR spectra were recorded from a dry film of the sample for the reaction control after emulsification.

2.2. pH measurements

The pH of the emulsion was determined right after the crosslinker was added and at different time intervals until the last polyaddition reaction was complete. All the determinations were carried out in a pH-meter HI 2211 pH/ORP-Meter (HANNA Instruments, Eibar, Spain) equipped with a pH electrode Crison 5029 (Crison Instruments, Barcelona, Spain) and a temperature probe.

2.3. Dynamic light scattering (DLS)

The size distribution of the NCs was analyzed on a Zetasizer Nano-ZS90 (Malvern, Worcestershire, UK) in Milli-Q water at 25 °C at a concentration of 0.5 mg/mL.

2.4. Zeta potential (Z-pot)

The Z-pot of the NCs was analyzed on a Zetasizer Nano-ZS90 (Malvern, Worcestershire, UK) in Milli-Q water at 25 °C at a concentration of 1 mg/mL, measured at different pH values.

2.5. Dialysis purification

The NCs were dialyzed against Milli-Q water for 24 h using a Spectra/Por molecular porous membrane tubing with a 12–14 kDa molecular weight cut-off (MWCO) (Spectrum Laboratories, Rancho Dominguez, USA).

2.6. Determination of cargo loading by UV-Vis spectroscopy

The coumarin and ICG loading (or dye-loading, DL) of the NCs was determined by UV-Vis measurements performed in a DINKO UV-6900 spectrophotometer (Dinko Instruments, Barcelona, Spain). First, a calibration curve was developed by preparing a range of standard solutions containing the coumarin at different concentrations and analyzing their UV profile and maximum absorbance (see Figure S14). Then, Encapsulation Efficiency EE (%) and Dye Loading DL (%) were calculated from the following equations:

$$\% \, EE = \frac{amount\ of\ dye\ incorporated\ in\ the\ nanocapsule}{total\ amount\ of\ dye\ added\ in\ the\ synthesis} * 100$$

$$\% \, DL = \frac{amount\ of\ dye\ incorporated\ in\ the\ nanocapsule}{total\ amount\ of\ dried\ nanocapsules} * 100$$

To determine the amount of coumarin incorporated in the NCs, a desired amount of dried NCs (previously dialyzed) was dissolved in an exact volume of solvent and the measurement was calculated from the calibration curve of free coumarin in the same organic solvent. To determine the amount of ICG incorporated in the NCs, a desired amount of suspended NCs (previously dialyzed) was diluted in an exact volume of water and the measurement was calculated from the calibration curve of free ICG in water. Non-loaded NCs were used as the reference. All measurements were recorded at the desired wavelength range, depending on the maximum of absorption of the molecule under study and assayed in triplicates.

2.7. Solids concentration

NCs concentration in the aqueous dispersion was determined by triplicate leading to dryness using a Digitheat-TFT oven (J.P.Selecta, Barcelona, Spain), with a fixed temperature of 40 ºC for 48 h.

2.8. Transmission electron microscopy (TEM)

The morphology of nanocapsules was studied on a Jeol JEM 1010 (Peabody, MA, USA). A 200-mesh copper grid coated with 0.75% FORMVAR was deposited on 6 μL of a suspension of nanocapsules in water (10 mg mL^{-1}) for 1 min. Excess of sample was removed by contact with ultrapure water for 1 min and the grid was deposited on a drop of uranyl acetate (2% w/w) in water for 1 min. Excess uranyl acetate was removed and the grid was air-dried for at least 3 h prior to measurement.

2.9 High-resolution transmission electron microscopy (HR-TEM)

HR-TEM observation was performed in a JEOL J2100 microscope (Peabody, MA, USA), operated at an accelerating voltage of 120 kV. Images were recorded using a Gatan Orius CCD camera. A 200-mesh copper grid coated with 0.75% FORMVAR was deposited on 6 μL of a suspension of nanocapsules in water (10 mg mL−1) for 1 min. Excess of sample was removed by contact with ultrapure water for 1 min and the grid was deposited on a drop of uranyl acetate (2% w/w) in water for 1 min. Excess uranyl acetate was removed and the grid was air-dried for at least 3 h prior to measurement.

3. Synthetic procedures

3.1. Synthesis of redox-responsive amphiphilic low HLB prepolymer (P1)

2,2´-Dihydroxyethyl disulfide (DEDS; yellow moieties) (2.86 g, 37.08 meq) and YMER N-120 (blue moieties) (3.64 g, 7.01 meq) were added into a three-necked round-bottom flask equipped with mechanical stirring at room temperature (rt) and purged with N_2. When the mixture was homogeneous, isophorone diisocyanate IPDI (black moieties) (8.83 g, 79.40 meq) was added into the reaction vessel under gentle mechanical stirring, checking the initial NCO stretching band intensity by FTIR. The polyaddition reaction was kept under these conditions until the NCO stretching band reach stabilization. At this point, dry acetone (18.2 mL) was added into the reaction mixture to fluidify the polymer. In parallel, 1,3-diamino-*N*-octadecylpropane (Genamin TAP 100D; red moieties) (9.25 g, 56.72 meq) was dissolved in dry acetone (8.1 mL) into another 100 mL three-necked round-bottom flask, which had previously been purged with N_2. The former reaction mixture was added dropwise onto the latter under half-moon 100 rpm mechanical stirring. The reaction was monitored by IR until the NCO stretching band intensity had completely disappeared.

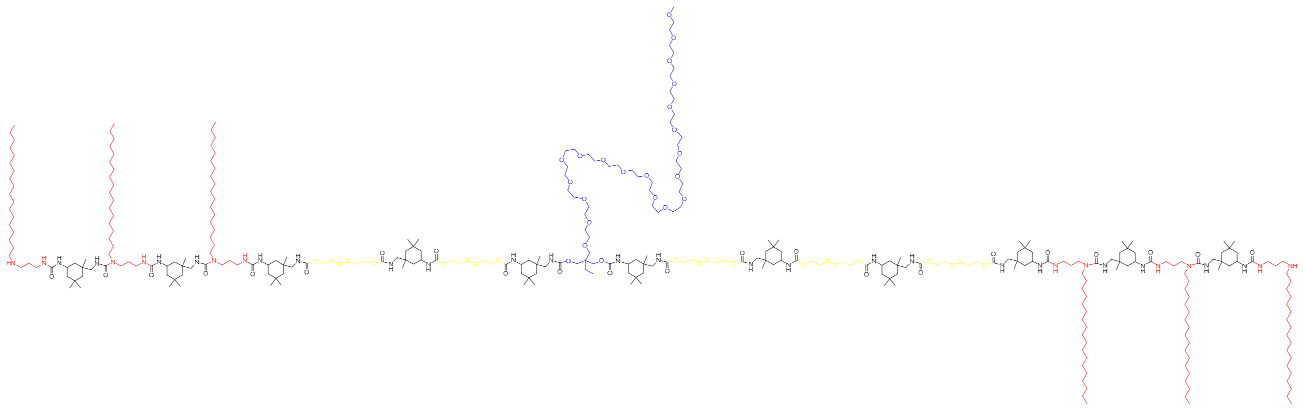

Figure S1: Structure of a representative amino-capped **P1** prepolymer.

3.2. Synthesis of redox responsive amphiphilic cationic prepolymer (P2)

2,2′-Dihydroxyethyl disulfide (DEDS; yellow moieties) (901.0 mg, 11.68 meq), YMER N-120 (blue moieties) (12.04 g, 23.18 meq) and *N*-(3-dimethylaminopropyl)-*N*,*N*′-diisopropanolamine (Jeffcat DPA; green moieties) (981.3 mg, 8.99 meq) were added into a three-necked round-bottom flask equipped with mechanical stirring at room temperature (rt) and purged with N_2. When the mixture was homogeneous, isophorone diisocyanate IPDI (black moieties) (8.14 g, 73.24 meq) was added into the reaction vessel under gentle mechanical stirring. The polyaddition reaction was kept under these conditions until the NCO stretching band intensity did not change, monitored by IR spectroscopy. At this point, dry THF (21 mL) was added into the reaction mixture to fluidify the polymer. In parallel, 1,3-diamino-*N*-octadecylpropane (Genamin TAP 100D; red moieties)) (5.99 g, 35.45 meq) was dissolved with dry THF (5.23 mL) into another 100 mL three-necked round-bottom flask, which had previously been purged with N_2. The former reaction mixture was added dropwise onto the latter under half-moon 100 rpm mechanical stirring. The reaction was monitored by IR until the NCO stretching band intensity had completely disappeared.

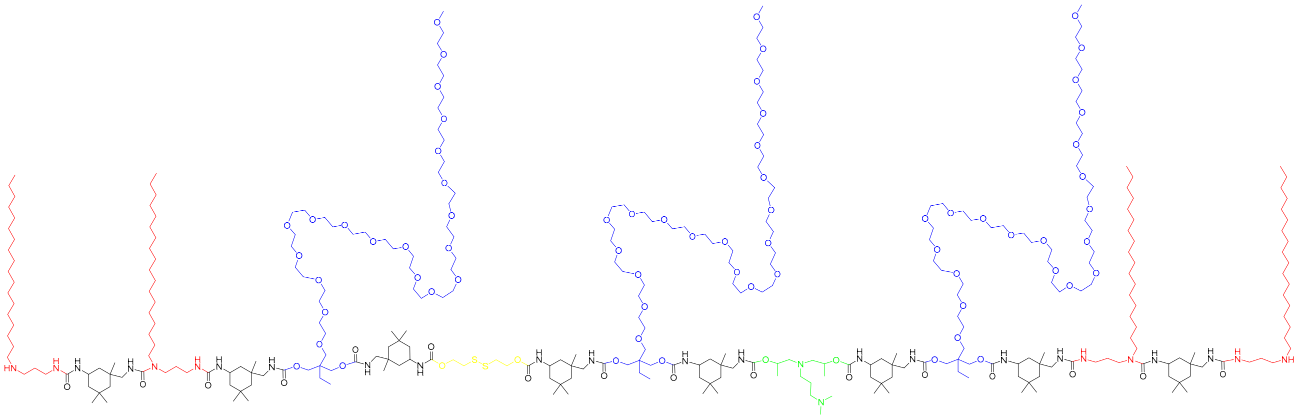

Figure S2: Structure of a representative amino-capped **P2** prepolymer.

3.3. Synthesis of dye-loaded cationic redox responsive water-in-oil-in-water NCs (cationic dye-NCs)

Table S1. Amounts of reagents used to prepare **cationic dye-loaded NCs.**

Substance	Amount	Equivalents or mols
**Dye**	5 mg	10.35 µmol COUPY or 6.35 µmol ICG
**IPDI**	265.0 mg	2.38 meq
**Cyclohexane**	1.28 mL	-
**Polymer in acetone (P1)**	635.2 mg	0.09 meq
**Polymer in THF (P2)**	5.87 g	0.61 meq
**Milli-Q water**	12.10 g	–
**DETA**	40.62 mg	1.18 meq

3.4. Synthesis of dye-loaded amphoteric redox responsive water-in-oil-in-water NCs (amphoteric dye-NCs)

Table S2. Amounts of reagents used to prepare **amphoteric dye-loaded NCs.**

Substance	Amount	Equivalents or mols
**Dye**	5 mg	10.35 µmol COUPY or 6.35 µmol ICG
**IPDI**	265.0 mg	2.38 meq
**Cyclohexane**	1.28 mL	-
**Polymer in acetone (P1)**	635.2 mg	0.09 meq
**Polymer in THF (P2)**	5.87 g	0.61 meq
**L-lysine**	0.65 mg	0.78 meq
**Milli-Q water**	12.88 g	–
**DETA**	13.93 mg	0.40 meq

3.5. Synthesis of non-loaded cationic and amphoteric redox responsive water-in-oil-in-water NCs (cationic and amphoteric control NCs)

Both cationic and amphoteric control nanocapsules were synthesized following the methodology described for dye-loaded ones, but without the initial addition of ICG or COUPY-derivate. Hence, the core of control nanocapsules only contains water.

3.6. Comparison of the structure of cationic and amphoteric ICG-loaded NCs

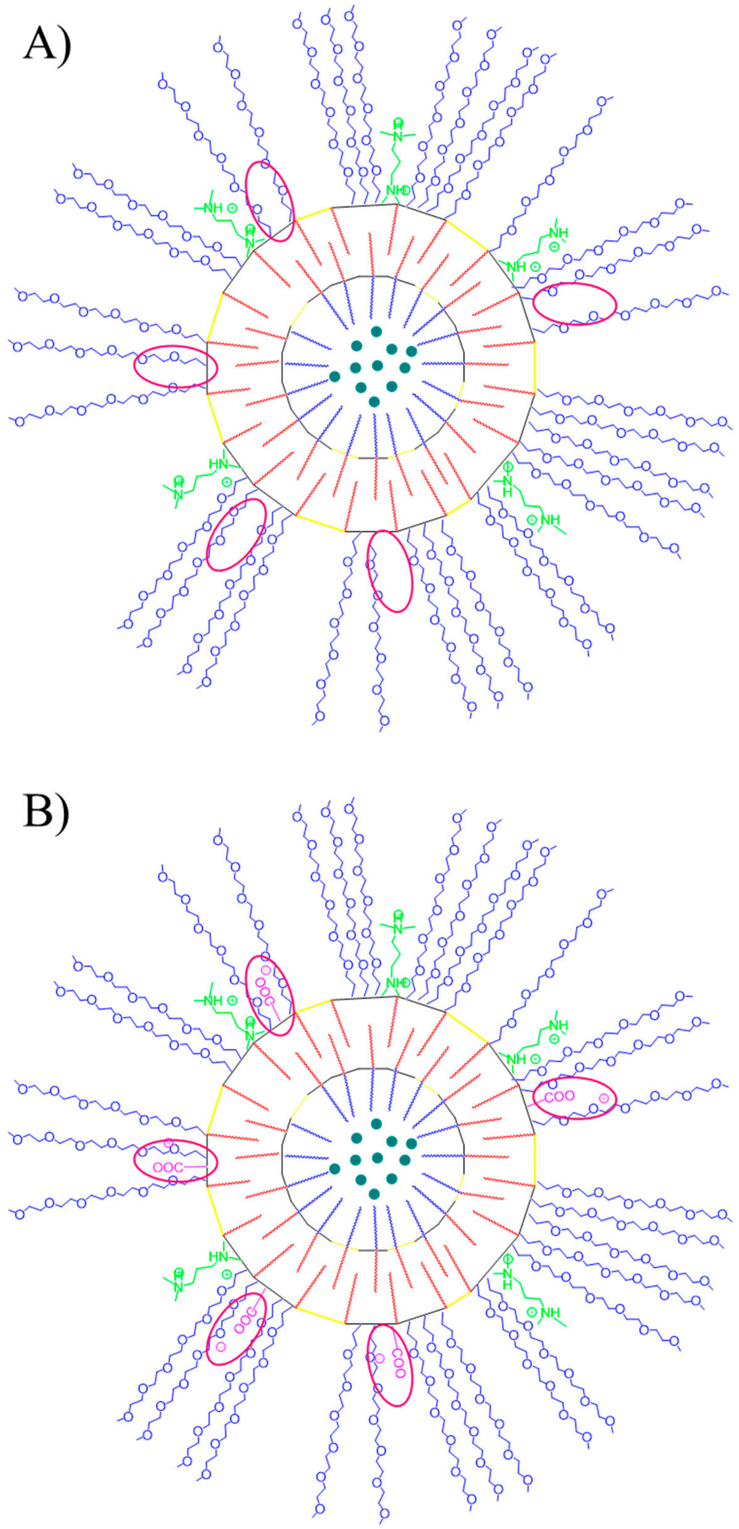

Figure S3. Comparison of the structure of A) cationic ICG-loaded NCs and B) amphoteric ICG-loaded NCs.

4. Characterization of polymers (P1 and P2) and NCs

4.1. Infrared Spectroscopy

The polymerization reaction was easily controlled by IR spectroscopy using the procedure detailed in section 2.1. given that NCO group has a very clear and characteristic stretching band at 2280-2230 cm^{-1}.

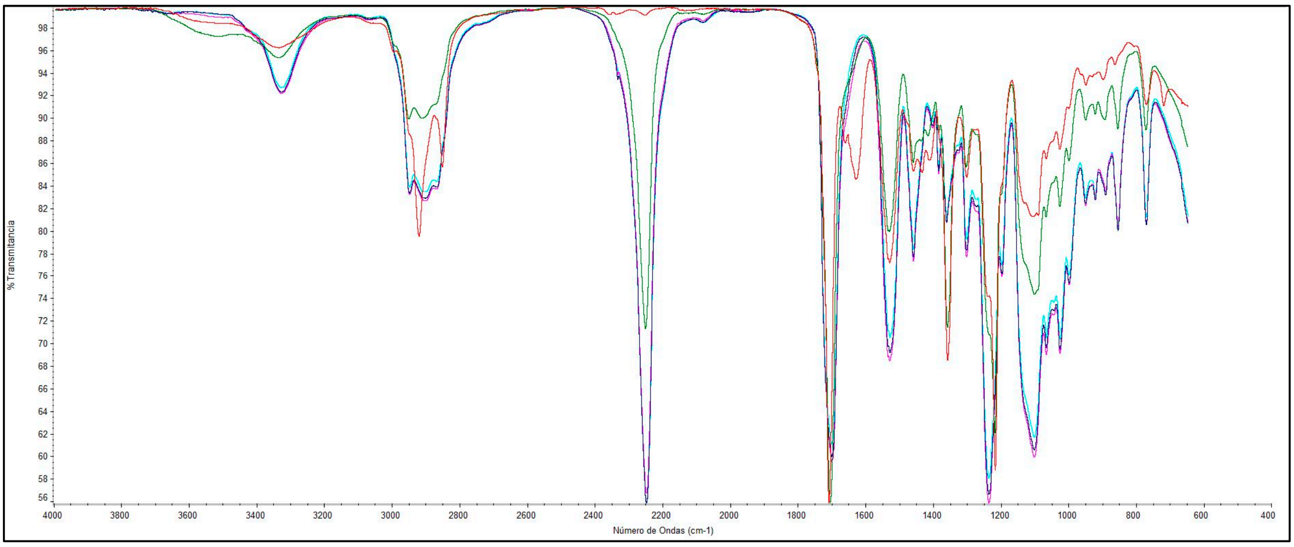

Figure S4. Consecutive IR spectra recorded during the synthesis of **P1**.

As shown in Figure S4, IR spectra of the polymer (**P1**) indicated a successful polymerization reaction between diols, the diamine and the diisocyanate, in both steps of the polymer synthesis. The purple line corresponds to the first sample recorded, at the begin of the reaction. At initial step of the reaction, the NCO asymmetric stretching band at 2252 cm^{-1} was very sharp and intense. After reacting with the diols, at the end of the first step (green line), the intensity of the NCO stretching band decreased significantly. Meanwhile, the intensities of the CO stretching band at 1719 cm^{-1}, the CN stretching band at 1537 cm^{-1} and the NCOO/COC asymmetric stretching band at 1240 cm^{-1} increased. Overall, the IR spectra registered during the first step of the synthesis confirmed polyurethane bond formation along with NCO consumption. Once the diamine was added during the second step of the polymer synthesis (red line), the NCO stretching band at 2252 cm^{-1} disappeared instantaneously, which was explained by the high reactivity of the amines. Simultaneously, other characteristic bands appeared or changed, such as a new stretching band at 1634 cm^{-1}, which was associated to the carbonyl of urea bonds and a new wagging band at 908 cm^{-1} corresponding to the free secondary amine, which also confirmed polyurea formation. Similar results were obtained during the synthesis of polymer **P2** (Figure S5).

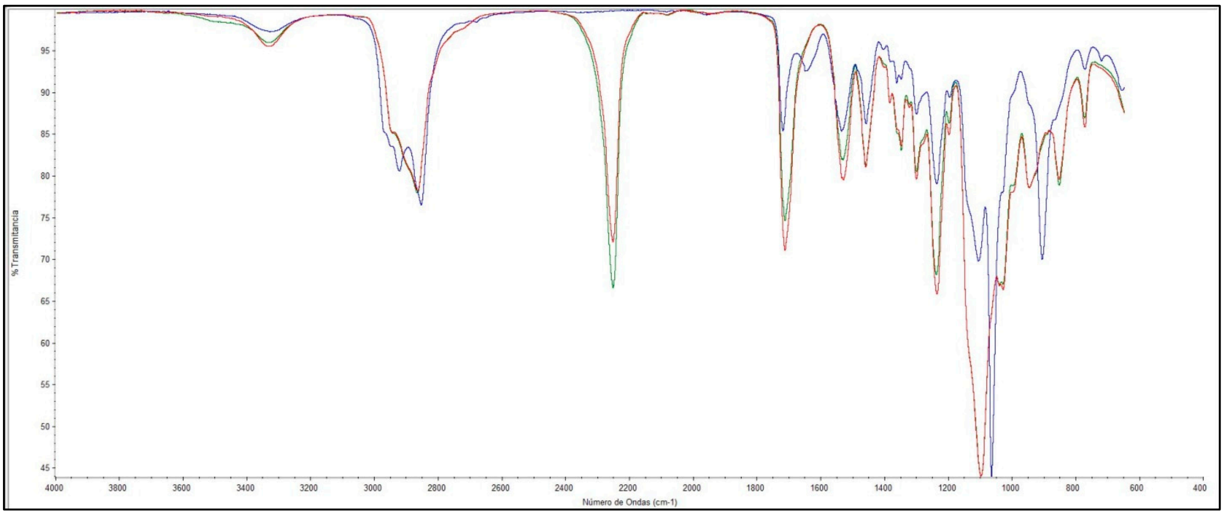

Figure S5. Consecutive IR spectra recorded during the synthesis of **P2**.

The encapsulation process was also controlled by IR spectroscopy. As shown in Figure S6, IR spectra of the water-in-oil encapsulation (regardless their loading) indicated also a successful nanocapsule formation, presenting small amount of free-NCO. In the next spectra (Figure S7) we can track the water-in-oil-in-water encapsulation process. The purple line corresponds to ENCAP 1 and P2 mixture, followed by the reactivation of the polymer with isocyanate and its conversion to an NCO-reactive entity (yellow line). Afterwards, L-lysine sodium salt was added (blue line), reacting with the activated polymer. A decrease on the intensity of the NCO stretching band at 2255 cm^{-1}, concomitantly with an increase of the carbonyl and CN stretching bands, confirmed urea formation (1642 cm^{-1} and 1532 cm^{-1}, respectively). The green line corresponds to a double-check of NCO presence after emulsion and, finally, the triamine was added (red line) and the NCO stretching band instantaneously disappeared by the same time that the urea-associated bands increased their intensity because of the rapid reaction between remaining NCO groups and this polyamine.

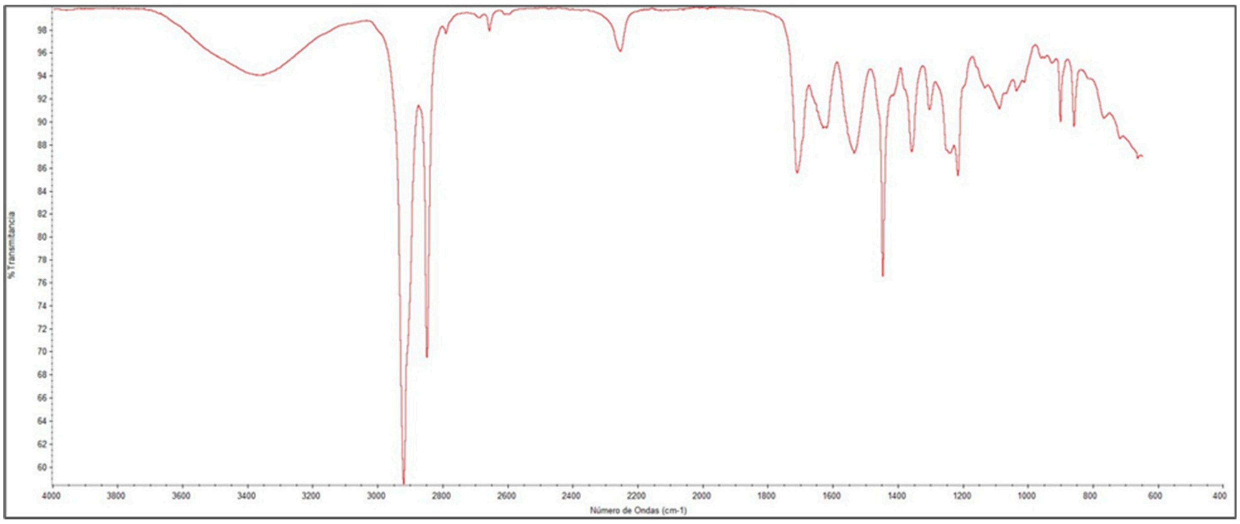

Figure S6. IR Spectrum of the W/O encapsulation reaction.

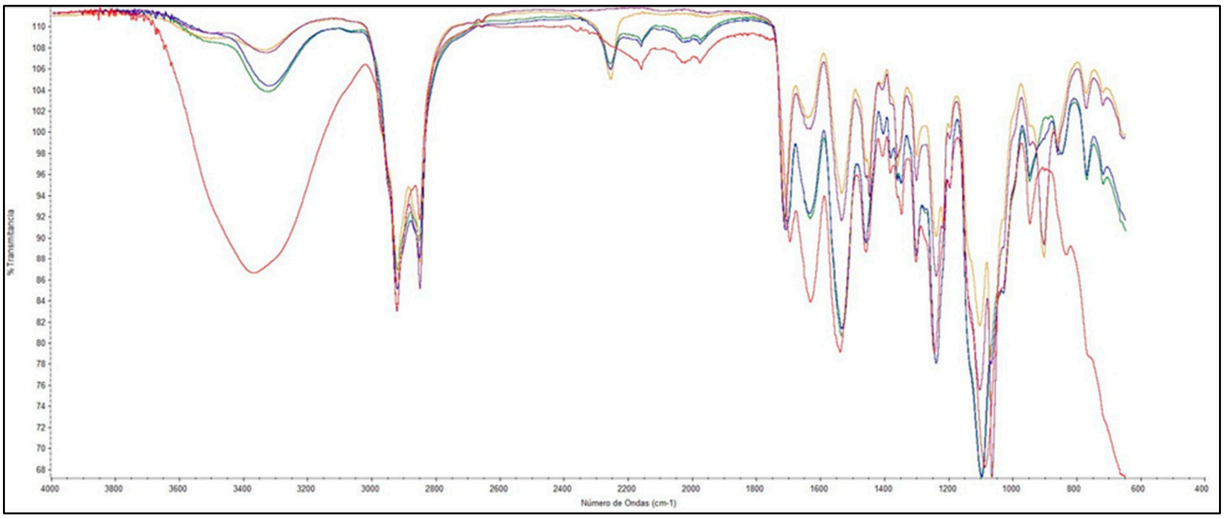

Figure S7. Consecutive IR spectra of the W/O/W encapsulation process.

4.2.Average size of NCs by Dynamic Light Scattering

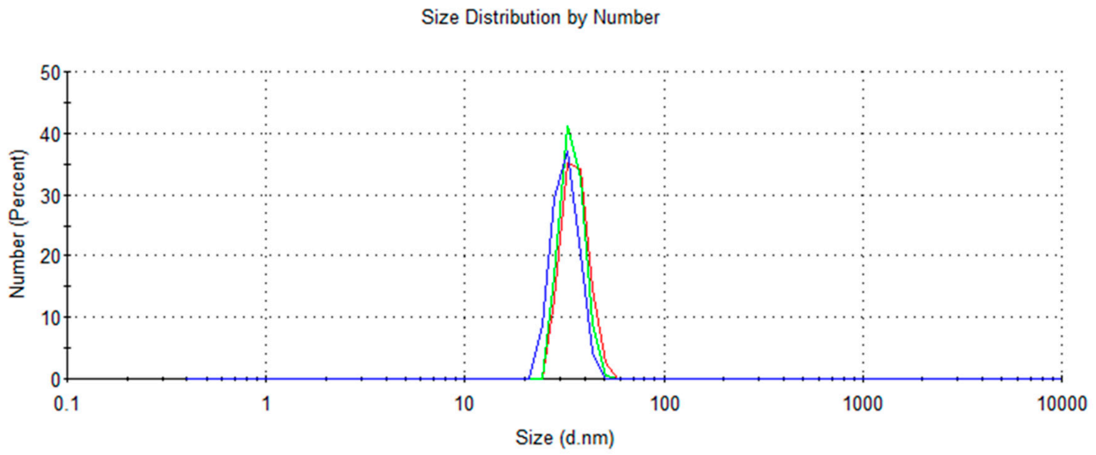

Figure S8. Hydrodynamic diameter distribution by number of cationic control NCs

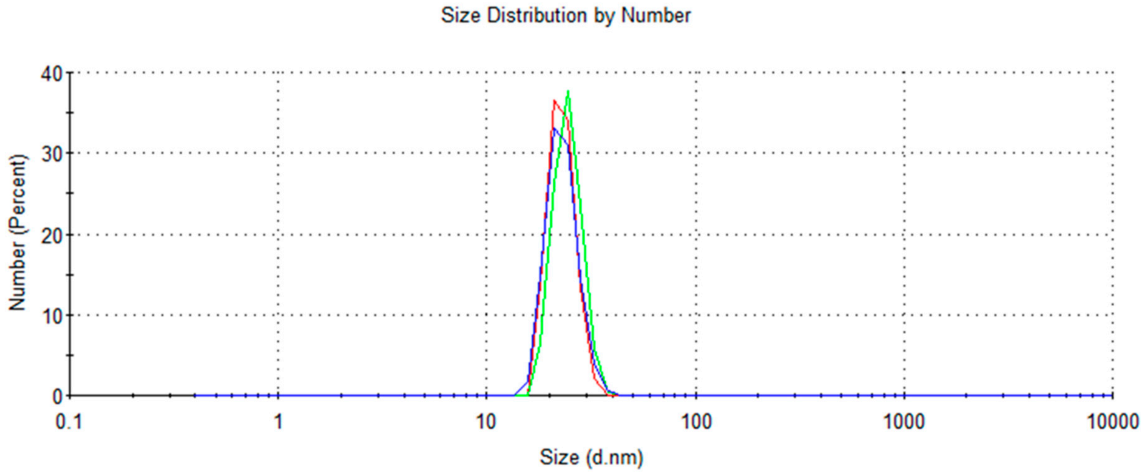

Figure S9. Hydrodynamic diameter distribution by number of amphoteric control NCs

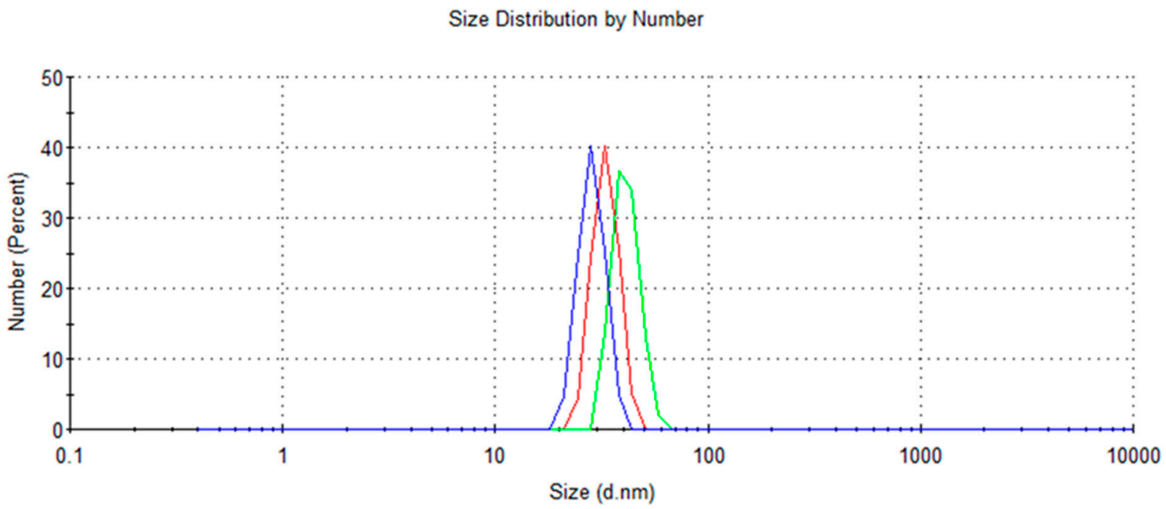

Figure S10. Hydrodynamic diameter distribution by number of cationic COUPY-NCs

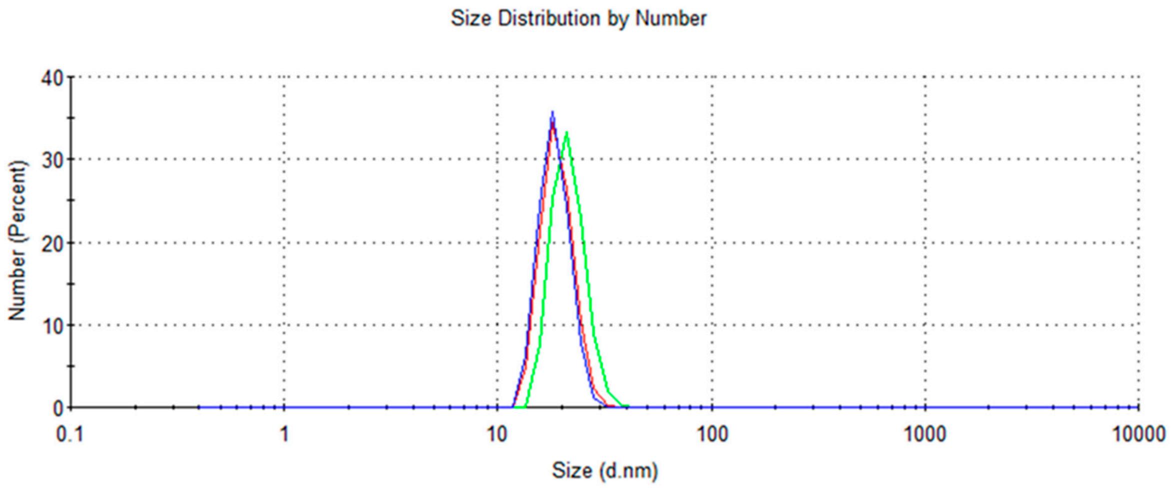

Figure S11. Hydrodynamic diameter distribution by number of amphoteric COUPY-NCs

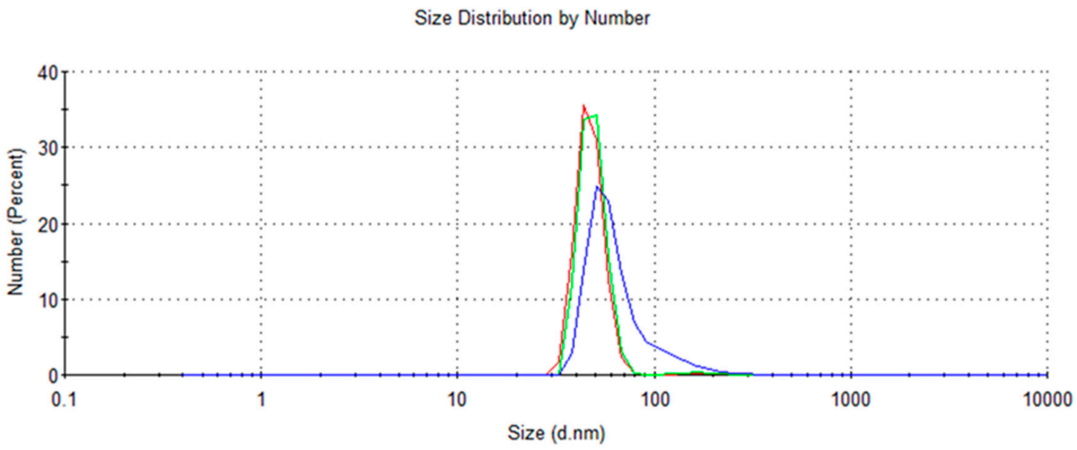

Figure S12. Hydrodynamic diameter distribution by number of cationic ICG-NCs

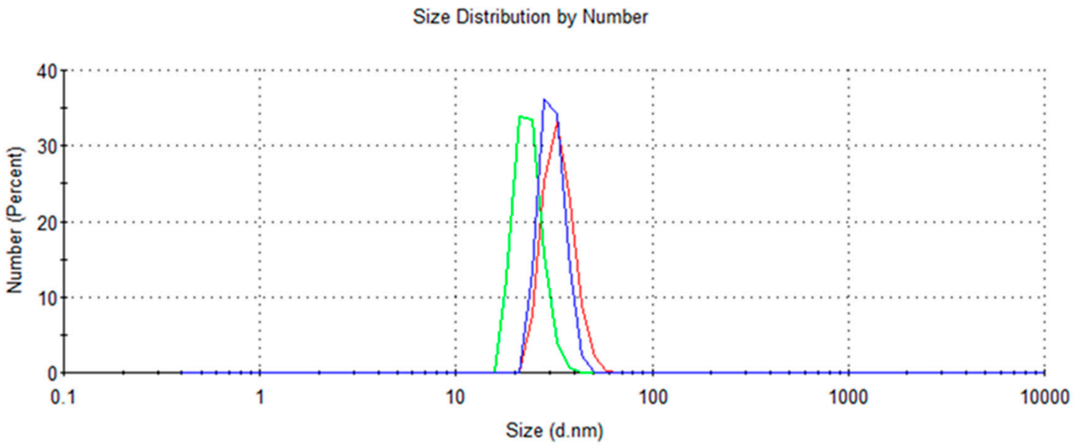

Figure S13. Hydrodynamic diameter distribution by number of amphoteric ICG-NCs

Table S3. DLS values obtained for the different types of NCs.

	Experimental Values	Average ± SD
**Cationic-Control-NCs**	36.36nm	34.55 ± 2.03 nm
	34.94 nm	
	32.36 nm	
**Amphoteric-Control-NCs**	23.03 nm	23.61 ± 0.82 nm
	24.55 nm	
	23.26 nm	
**Cationic-COUPY-NCs**	33.33 nm	34.59 ± 6.60 nm
	41.73 nm	
	28.71 nm	
**Amphoteric-COUPY-NCs**	19.09 nm	19.74 ± 1.61 nm
	21.58nm	
	18.56 nm	
**Cationic-ICG-NCs**	48.28 nm	55.22 ± 10.42 nm
	50.17 nm	
	67.20 nm	
**Amphoteric-ICG-NCs**	33.63 nm	29.37 ± 5.25 nm
	23.50 nm	
	30.98 nm	

4.3 ζ -potential of non-loaded NCs

ζ -potential (mV)	Experimental values			
	**pH = 6.5**	**pH = 7.0**	**pH = 7.5**	**pH = 8.0**
**Cationic non-loaded NCs**	50.2	47.9	36.0	32.9
	48.6	47.9	34.1	31.3
	48.3	48.2	34.5	33.8
**Amphoteric non-loaded NCs**	25.8	18.1	12.2	4.2
	26.2	18.2	12.0	4.33
	27.1	19.1	12.2	4.05

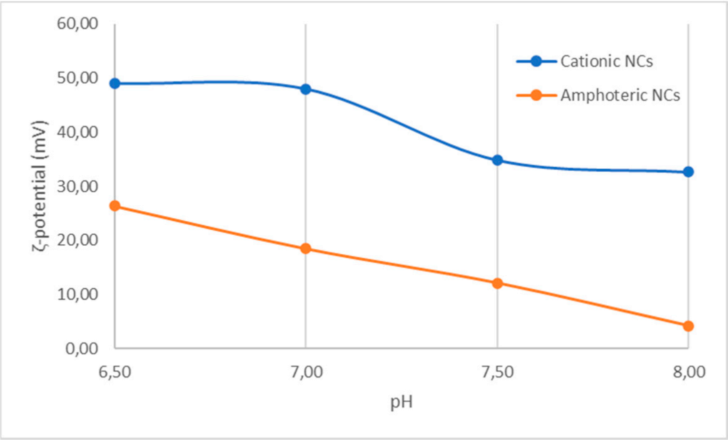

Figure S14. Plot of ζ-potential mean values of both cationic and amphoteric non-loaded NCs vs pH

4.4. ICG and COUPY payload determination in NCs

The concentration of NCs in the final emulsions (mg/mL) was quantified with a solids concentrator, as indicated in section 2.7. Dye Loading (DL) and Encapsulation Efficiency (EE) were determined by UV-Visible spectroscopy following the method described in section 2.6. The calibration plot for COUPY and ICG, determined at 567 nm and at 794 nm, respectively, is shown in Figure S14. ICG was dissolved in water and the COUPY derivative in 1% DMSO in water. DL and EE parameters for NC-COUPY and NC-ICG are provided in Table S4.

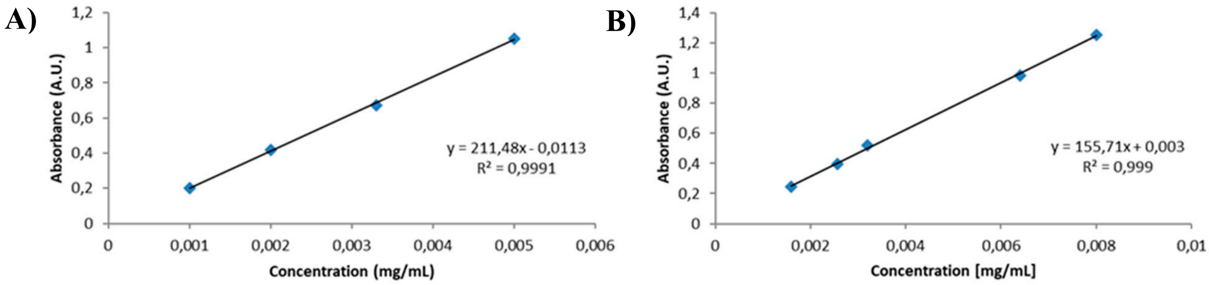

Figure S15. Calibration plot for A) COUPY and B) ICG.

Table S4. NCs concentration, DL and EE parameters for NCs.

	[NCs] (mg/mL)	Dye Loading (DL, µM)	Dye Loading per mL of emulsion (DL, mg/mL)	Dye Loading per g of nanocapsule (DL, mg dye/g NCs)	Encapsulation Efficiency (EE, %)
**Non-loaded cationic NCs**	137.21 ± 1.31	-	-	-	-
**Non-loaded amphoteric NCs**	150.00 ± 2.00	-	-	-	-
**Cationic COUPY-NCs**	173.46 ± 1.16	482.99 ± 1.25	0.37	2.16	42.24%
**Amphoteric COUPY-NCs**	154.92 ± 2.09	470.46 ± 0.96	0.36	2.35	44.62%
**Cationic ICG-NCs**	199.03 ± 2.00	215.49 ± 2.58	0.17	0.84	53.91%
**Amphoteric ICG-NCs**	134.75 ± 0.67	237.42 ± 3.50	0.18	1.37	61.91%

4.5. TEM analysis

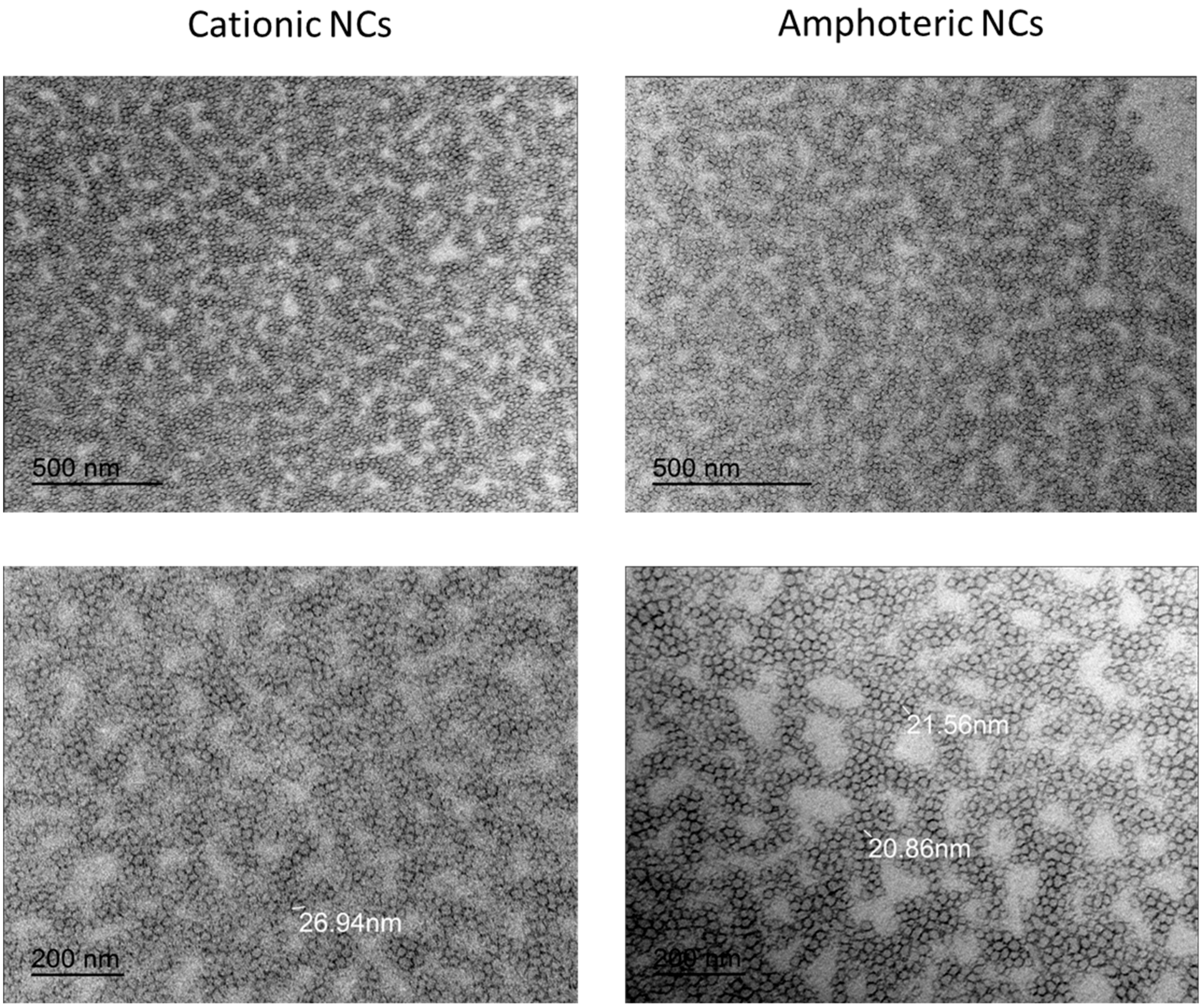

Figure S16. TEM micrographs of cationic and amphoteric control NCs

4.6 Stability of amphoteric ICG-loaded NCs under different conditions

The stability of the fluorescent probe, either free or nanoencapsulated, was evaluated in PBS buffer by UV-vis spectroscopy after subjected to three different experimental conditions during 1 week: 1) fridge storage at 4 ºC in the darkness, 2) room temperature (RT) storage under ambient light, and 3) oven incubation at 37 ºC in the darkness. The absorbance of each sample has been normalized against the maximum absorbance at the starting point of the experiment.

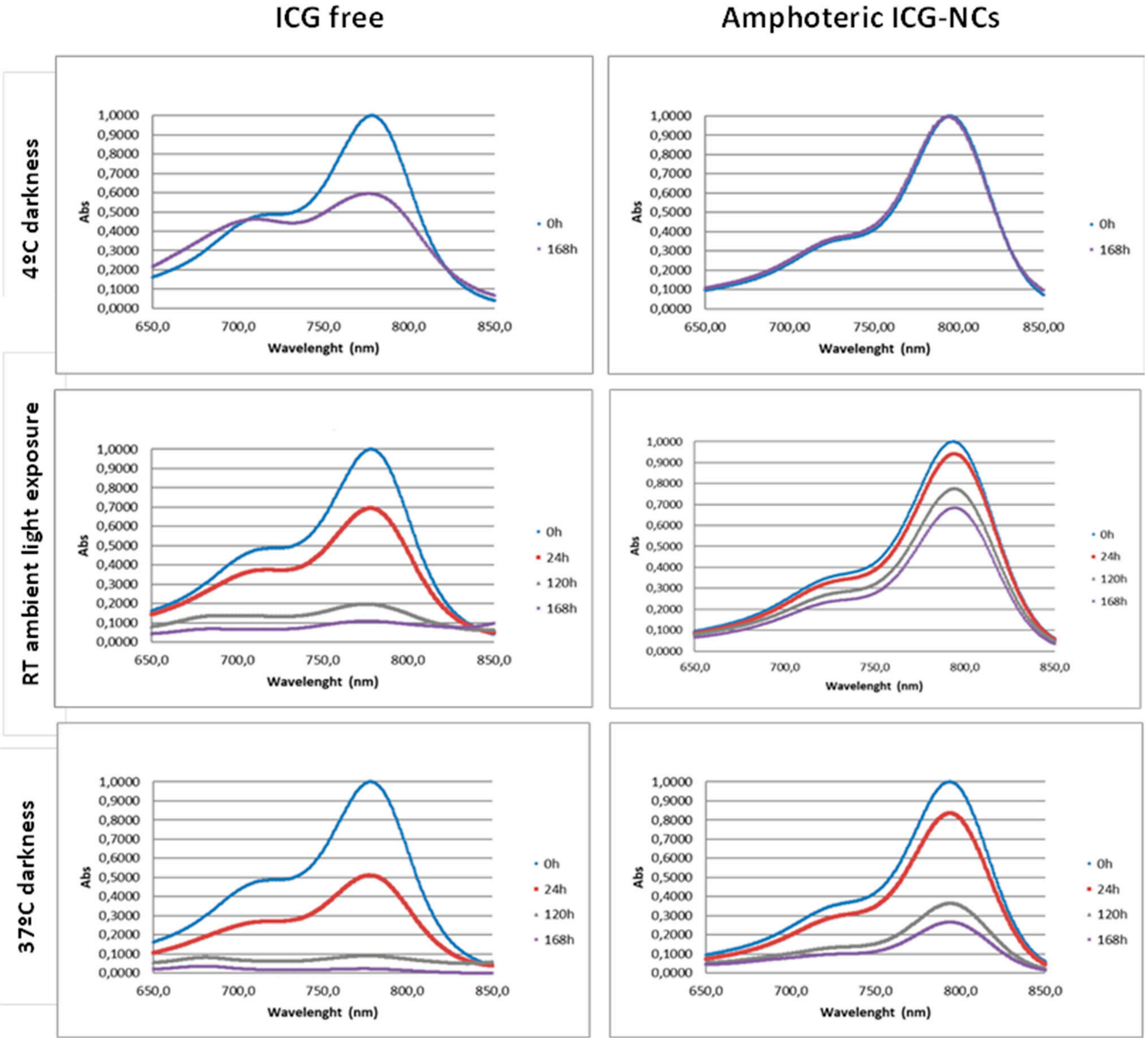

Figure S17. Normalized UV-visible absorption spectra of ICG free and amphoteric ICG-loaded NCs after 1 week (168 h) under different experimental conditions: 1) Fridge storage at 4 ºC in the darkness, 2) Room temperature (RT) storage at ambient light exposure and 3) incubation at 37 ºC in the darkness.

5. Biological studies

5.1. Internalization evaluation of ICG-loaded NCs in human monocyte derived dendritic cells (moDCs) by confocal microscopy

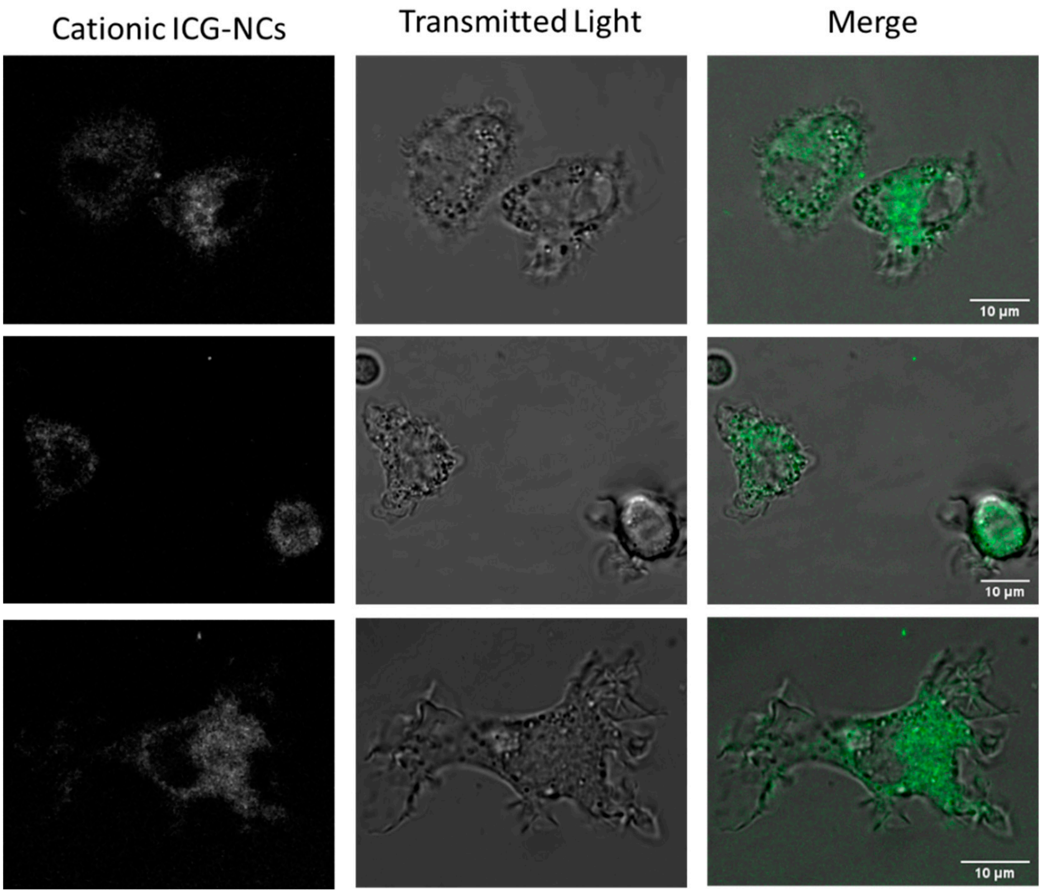

Figure S18. Internalization pattern for cationic NCs-ICG at 2 µM of ICG in moDCs for 2 h incubation at 37 ºC. Scale bar represents 10 µm.

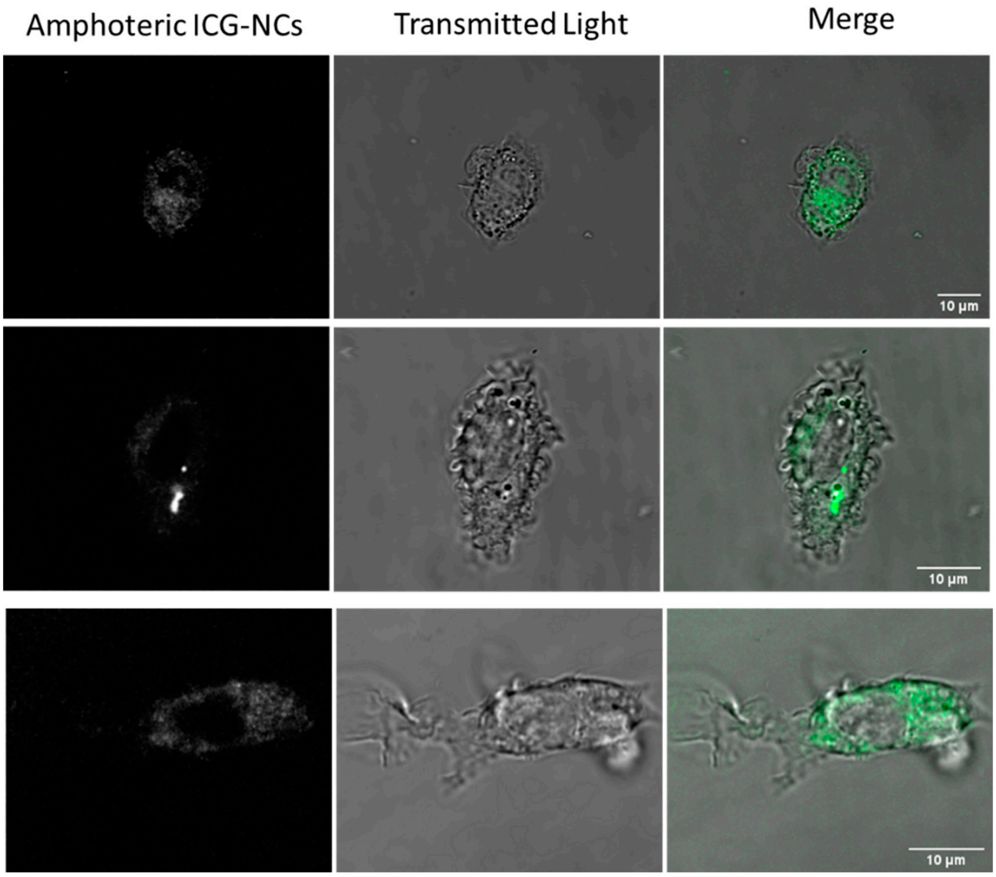

Figure S19. Internalization pattern for amphoteric NCs-ICG at 2 μM of ICG in moDCs for 2 h incubations at 37 ºC. Scale bar represents 10 μm.

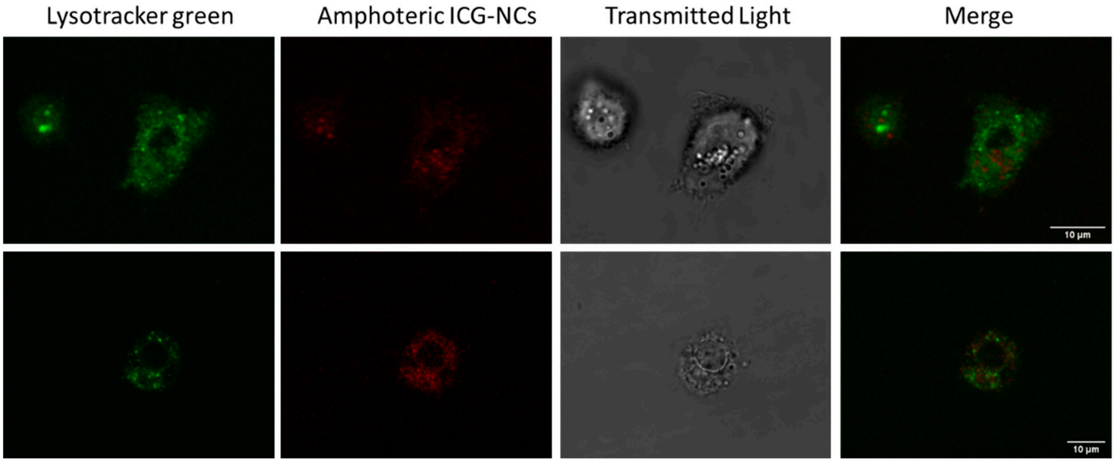

Figure S20. Colocalization pattern of amphoteric ICG-NCs and Lysotracker Green in moDCs. Incubation for 2 h at 37 ºC.

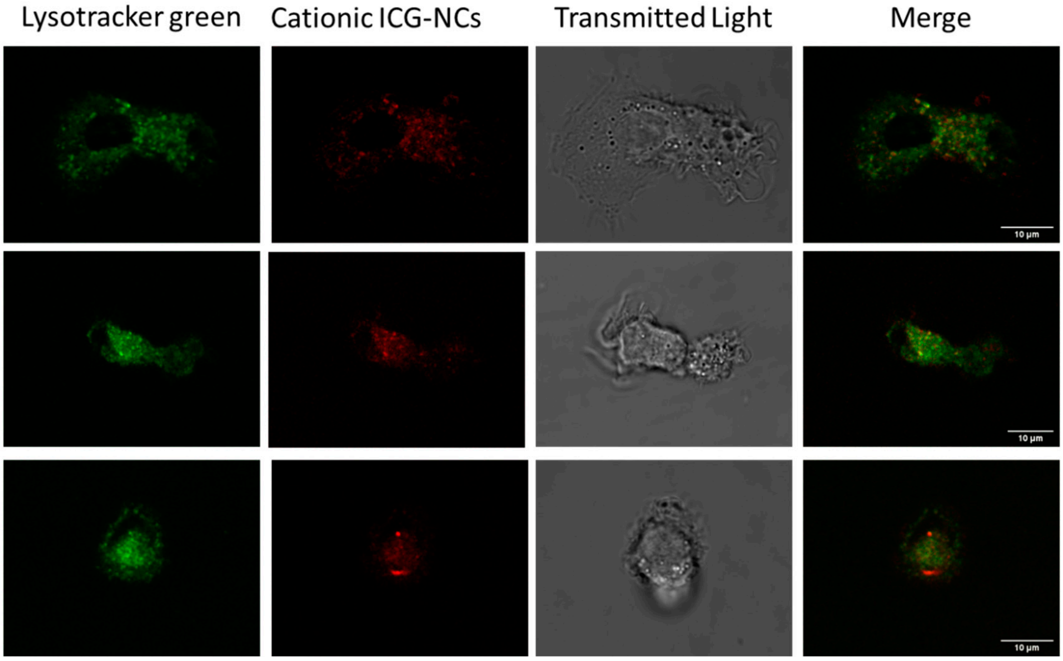

Figure S21. Colocalization pattern of cationic ICG-NCs and Lysotracker Green in moDCs. Incubation for 2 h at 37 ºC.

5.2. *In vitro* Internalization studies in human monocyte derived immature DCs by Flow Cytometry

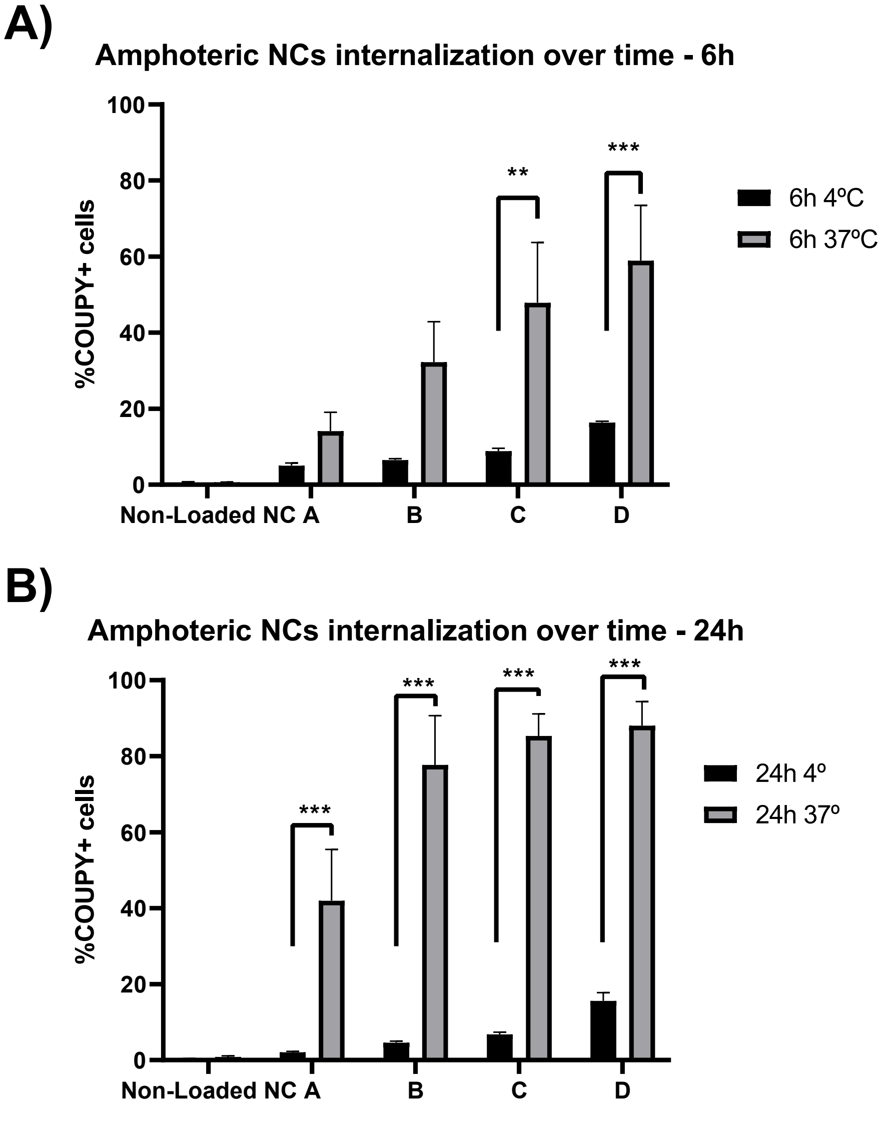

Figure S22. COUPY-loaded amphoteric nanoparticles internalization over time: A) 6h incubations and B) 24 h incubations. Conditions A-D correspond to NC concentrations of: A) 1.24 µL/mL, B) 3.105 µL/mL, C) 6.21 µL/mL, D) 12.42 µL/mL and Non-loaded NC) 1.,61 µL/mL. The graphic depicts percentages of COUPY-positive DCs from n=3 independent experiments. Significance was determined by unpaired T-tests assuming an equal scatter amongst samples using the Holm-Sidak method. p value outputs are in APA style (.12(ns), .33(*), .002(**), <.001(***)). Two-way ANOVA tests and Sidak's multiple comparisons test with a single pooled variance determined no statistical significance among 4ºC samples, and for 37 ºC samples a p=.002(**) between conditions A-D (A), a p=.002(**) between conditions A-B and A-C (B) and a p=<.001(***) between conditions A-D (B). Flow Cytometer: Attune NxT from ThermoFischer.

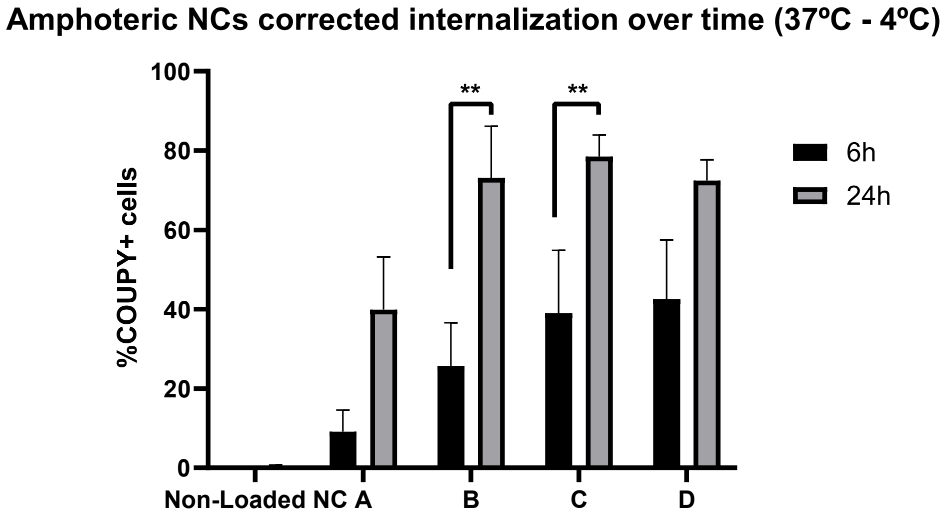

Figure S23. COUPY-loaded amphoteric NCs corrected internalization. 6 h versus 24 h incubations. 4 ºC positive percentages have been subtracted from 37 ºC ones to correct for membrane bound NCs. Conditions A-D correspond to NC concentrations of: A) 1.24 µL/mL, B) 3.105 µL/mL, C) 6.21 µL/mL, D) 12.42 µL/mL and Non-loaded NC) 1.61 µL/mL. Graphic depicts n=3 experiments. Significance was determined by unpaired T-tests assuming an equal scatter amongst samples using the Holm-Sidak method. p value outputs are in APA style (.12(ns), .33(*), .002(**), <.001(***)). Flow Cytometer: Attune NxT from ThermoFischer.

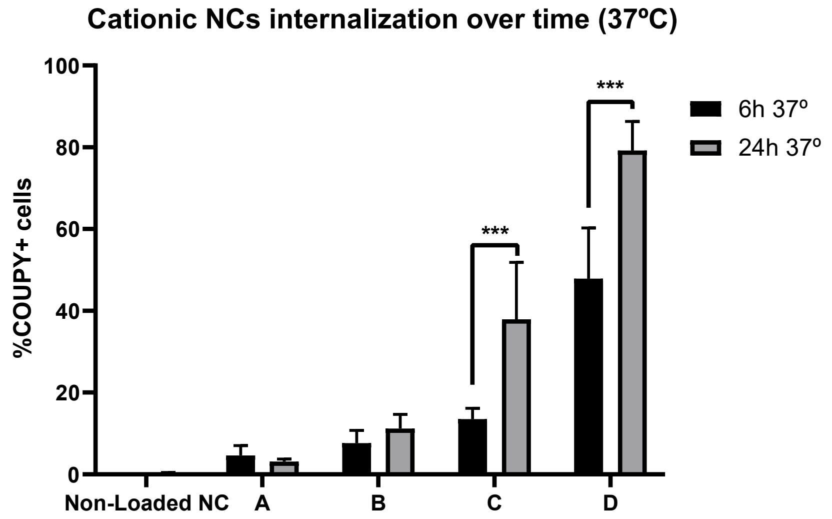

Figure S24. COUPY-loaded cationic NCs internalization over time. 6 h versus 24 h incubations at 37ºC. Conditions A-D correspond to NC concentrations of: A) 0.619 µL/mL, B) 1.55 µL/mL, C) 3.1 µL/mL, D) 6.19 µL/mL and Non-loaded NC) 3.29 µL/mL. Graphic depicts n=4 independent experiments. Significance was determined by unpaired T-tests assuming an equal scatter amongst samples using the Holm-Sidak method. p value outputs are in APA style (.12(ns), .33(*), .002(**), <.001(***)). Flow Cytometer: Attune NxT from ThermoFischer.

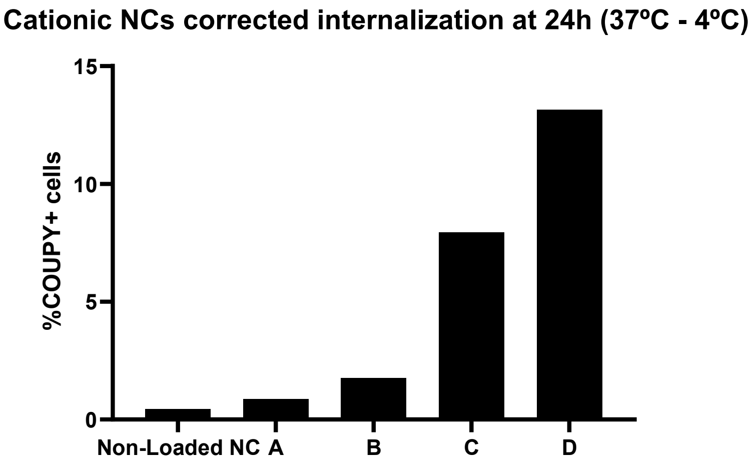

Figure S25. COUPY-loaded cationic NCs internalization at 24 h incubations. 4º positive percentages have been subtracted from 37ºC ones to correct for membrane bound NCs. Conditions A-D correspond to NC concentrations of: A) 0.619 µL/mL, B) 1.55 µL/mL, C) 3.1 µL/mL, D) 6.19 µL/mL and Non-loaded NC) 3.29 µL/mL. Graphic depicts n=1 independent experiments. Flow Cytometer: Attune NxT from ThermoFischer.

5.3 *In vivo* safety assay of amphoteric and cationic control NCs

Table S5. Hematological parameters in blood (Mean ± SD)

Indexes	*Control*	*Cationic control NCs*			*Amphoteric control NCs*		
**Concentration**		low	medium	high	low	medium	high
**WBC (10^3/uL)**	3,31±0,02	3,12±0,51	4,14±1,09	2,49±0,18	3,63±0,87	3,57±1,15	2,79±0,29
**RBC (10^6/uL)**	10,76±0,48	10,43±0,05	10,5±0,45	11,22±0,08	10,45±0,04	10,63±0,11	10,82±0,09
**HGB (g/dL)**	16,2±1,27	15,9±0	15,86±0,81	17,06±0,32	15,85±0,21	16,03±0,2	16,43±0,05
**HCT (%)**	47±2,54	45,76±0,25	46,2±2,42	49,1±0,75	45,7±0,14	46,7±0,52	47,66±0,15
**MCV (fL)**	43,7±0,42	43,9±0,36	44±0,55	43,76±0,4	43,75±0,35	43,93±0,23	44,06±0,45
**MCH (pg)**	15,05±0,49	15,23±0,05	15,06±0,23	15,2±0,17	15,2±0,28	15,1±0,09	15,16±0,15
**MCHC (g/dL)**	34,4±0,84	34,73±0,15	34,33±0,15	34,76±0,3	34,65±0,35	34,33±0,05	34,46±0,05
**PLT (10^3/uL)**	880,5±137,9	876,3±54,6	652,7±399,2	1005,3±102	798,5±217,1	962,7±293,9	784,3±224,1
**LYMPH (%)**	79±1,27	68,43±9,06	59,43±1,96	53,93±10,05	63,85±6,15	59,73±6,65	31,2±25,63
**MONO (%)**	1,65±0,21	1,1±0,6	1,63±0,23	2±0,55	1,15±0,63	0,96±0,6	1,9±0,43
**NEUT (%)**	17,25±2,33	27,73±8,97	30,7±1,44	38,16±6,29	28,75±0,35	29,36±3,49	56,5±18,55
**EO (%)**	2,1±0,84	2,73±1,01	8,16±3,32	5,9±4,24	6,1±6,92	9,93±6,83	10,4±8,11
**BASO (%)**	0±0	0±0	0,06±0,11	0±0	0,15±0,21	0±0	0±0

Table S6. Biochemical parameters in serum (Mean ± SD)

Indexes	*Control*	*Cationic control NCs*			*Amphoteric control NCs*		
**Concentration**		low	medium	high	low	medium	high
**Body weight**	1,35±1,17	0,69±1,2	0,81±1,41	0,5±0,87	1,5±2,6	1,15±1,23	1,85±1,66
**Albumin (g/dL)**	2,87±0,06	2,59±0,24	2,49±0,09	3,31±1,09	2,51±0,17	2,61±0,28	2,44±0,71
**ALT (U/L)**	61,06±22,8<br>1	61,73±30,6<br>9	42,3±12,47	87,33±81,12	60,05±47,2	42,4±24,97	42,45±12,51
**AST (U/L)**	930±132,8	785,13±305	915,2±123,2	1103,46±702	577±172,5	792,16±92,6	600,76±295
**TBIL (mg/dL)**	<0.02	<0.02	<0.02	<0.02	<0.02	<0.02	<0.02
**CREA (mg/dL)**	0,18±0,06	0,22±0,06	0,27±0,03	0,17±0,03	0,2±0,03	0,21±0,03	0,5±0,46
**GGT (U/L)**	< 1	< 1	< 1	< 1	< 1	< 1	< 1
**Potassium (mmo/L)**	7,37±0,3	5,36±0,23	6,17±0,84	9,64±3,67	9,61±4,16	6,81±1,38	6,25±1,48
**Total proteins (g/dL)**	5,04±0,48	4,38±0,49	4,29±0,22	5,54±1,47	4,51±0,31	4,56±0,38	4,35±1,27
**Sodium (mmol/L)**	151,2±0,6	147,6±2,26	143,56±2,05	145,26±5,97	141,36±4,8	143,36±3,09	154,03±5,68
**Urea (mg/dL)**	41,17±6,8	37,93±4,96	33,99±4,66	38,69±6,24	32,69±0,57	30,98±1,44	169,48±236

5.4. Biodistribution of amphoteric and cationic ICG-loaded NCs in mice models.

5.4.1 *In vivo* fluorescence imaging biodistribution in healthy mice.

5.4.1.1 Intravenous administration in C57BL/6 mice.

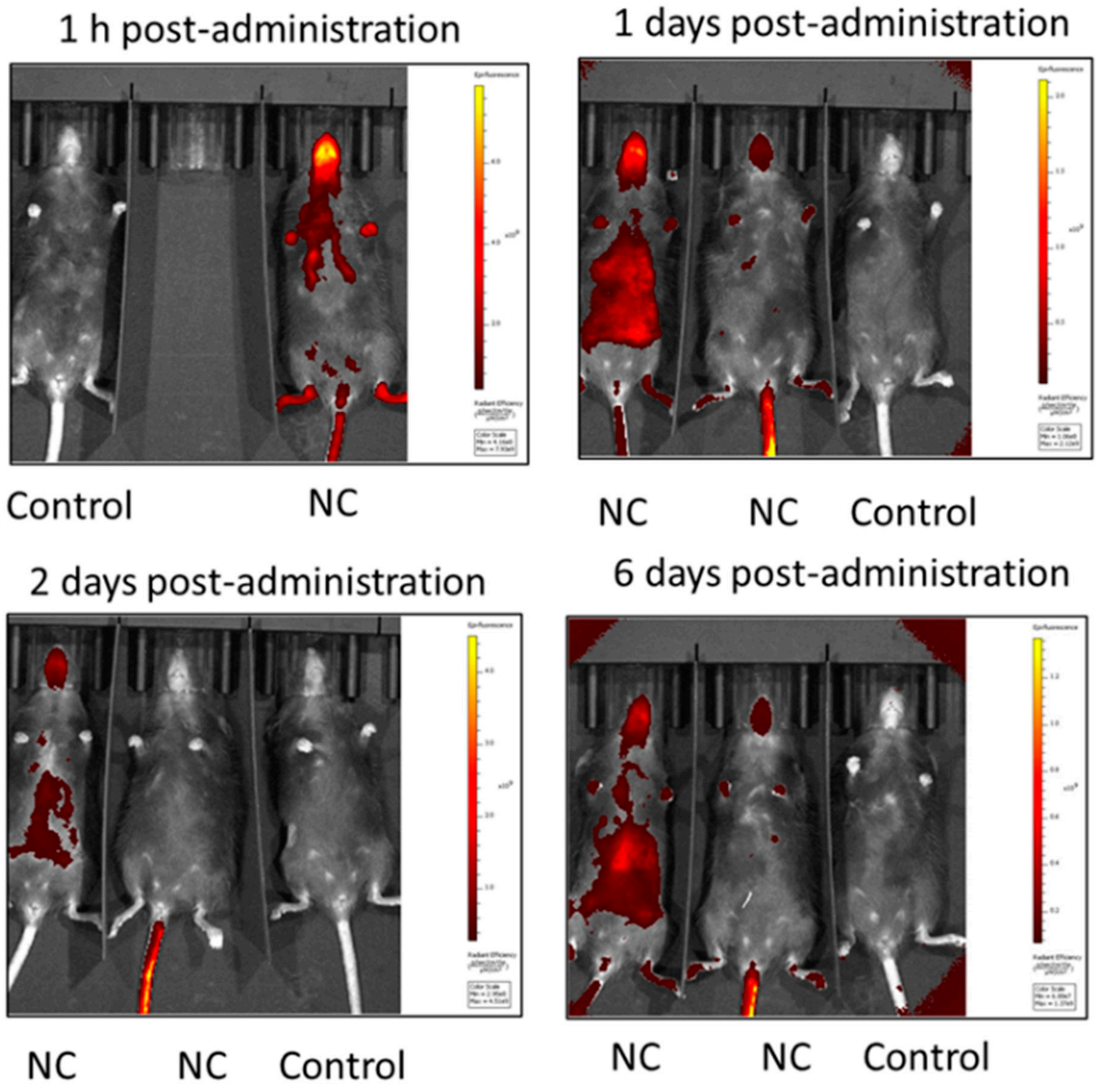

Figure S26. Cationic ICG-NCs (200 µL, 40 µM ICG) were intravenously administrated to female C57BL/6 mice (n=2). Fluorescence was monitoring by *in vivo* imaging system (IVIS; Perkin Elmer, Waltham, MA, USA,) using excitation/emission wavelengths of 780 nm/845 nm

5.4.1.2 Comparison on excretion organs NCs signal between different administrations in C57BL/6 mice model

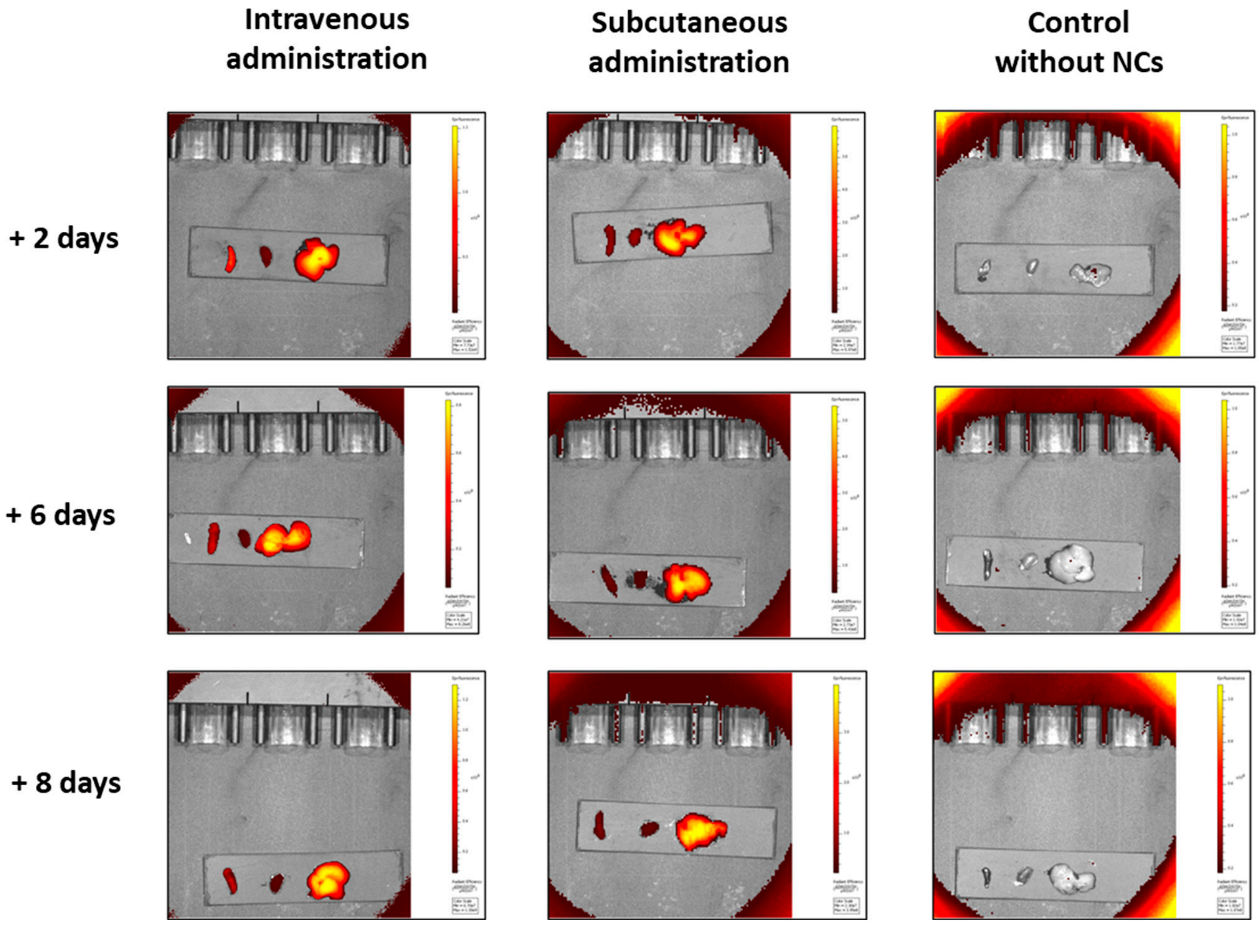

Figure S27. Cationic ICG-NCs (200 µL, 40 µM of ICG) were intravenously or subcutaneously administrated to female C57BL/6 mice (n=2/group). At indicated time points mice were euthanized and spleen (left), kidney (middle) and liver (right) were obtained for fluorescence monitoring by *in vivo* imaging system (IVIS; Perkin Elmer, Waltham, MA, USA,) using excitation/emission wavelengths of 780 nm/845 nm.

5.4.1.3 Intravenous administration biodistribution comparison between ICG, amphoteric ICG-NCs and cationic ICG-NCs at 1 h, 24 h and 48 h in healthy BALB/C mice model

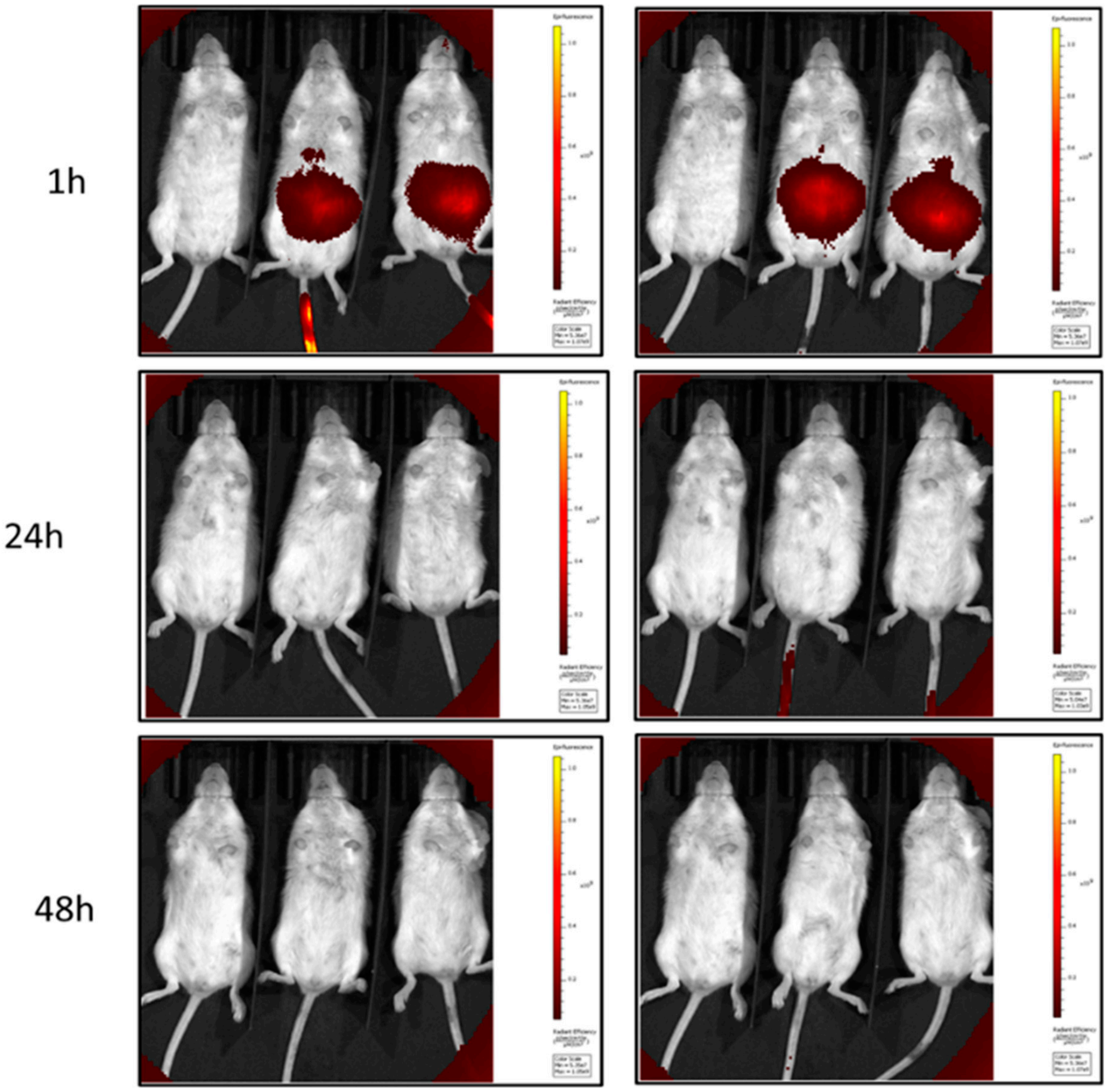

Figure S28. Intravenous administration of 160 µl, 40 µM of free **ICG**, on BALB/C mice group (n=4 mouse. Control mouse located in the left-side of each image.

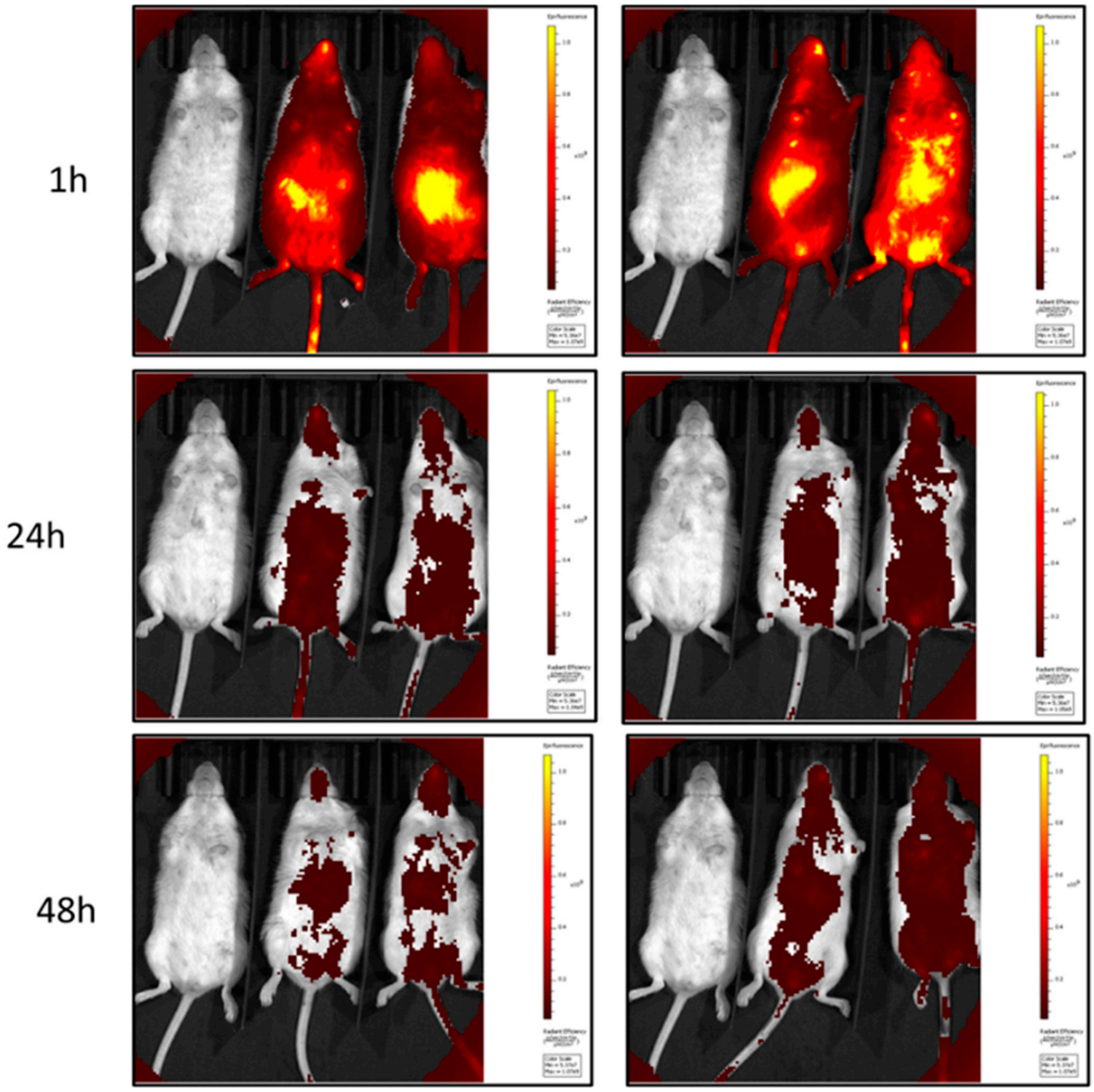

Figure S29. Intravenous administration of 160 µl, 40 µM of ICG in 30 mg/mL of cationic ICG-NCs, on BALB/C mice group (n=4 mouse). Control mouse located in the left-side of each image.

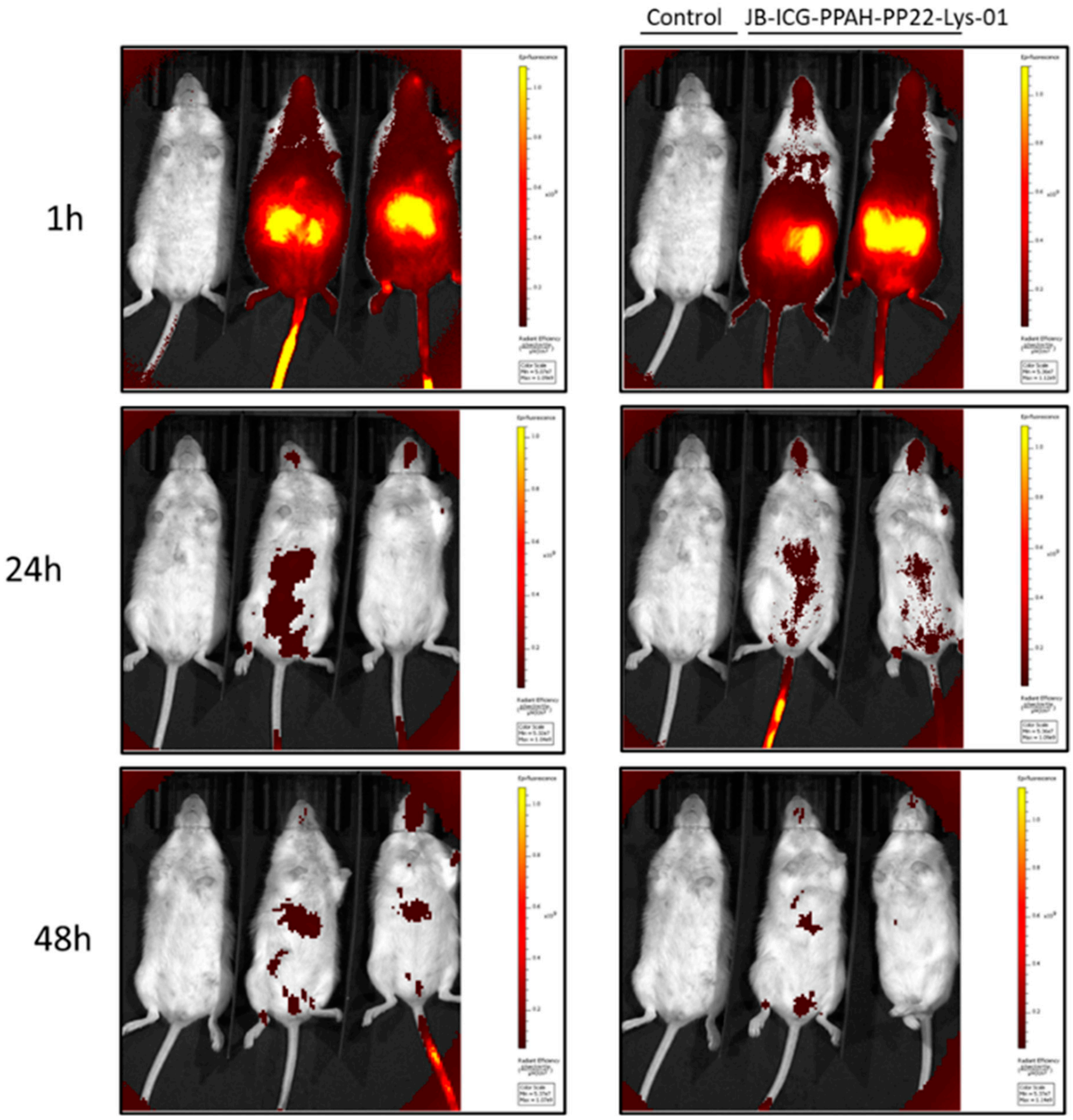

Figure S30. Intravenous administration of 160 µl, 40 µM of ICG in 30 mg/mL of amphoteric ICG-NCs, on BALB/C mice group (n=4 mouse). Control mouse located in the left-side of each image.

5.4.2 *In vivo* fluorescence imaging biodistribution in subcutaneous tumor mice models.

Female NSG mice (9 weeks old, Charles River) were challenged by s.c. injection of A375 (1×106) cells on the right flank with a 23-gauge needle. Amphoteric ICG-NCs and cationic ICG-NCs were administered i.v. at day 14 post-tumor challenge. PBS and ICG free were injected to control groups. Fluorescence was monitoring by *in vivo* imaging system (IVIS; Perkin Elmer, Waltham, MA, USA,) using excitation/emission wavelengths of 780 nm/845 nm.

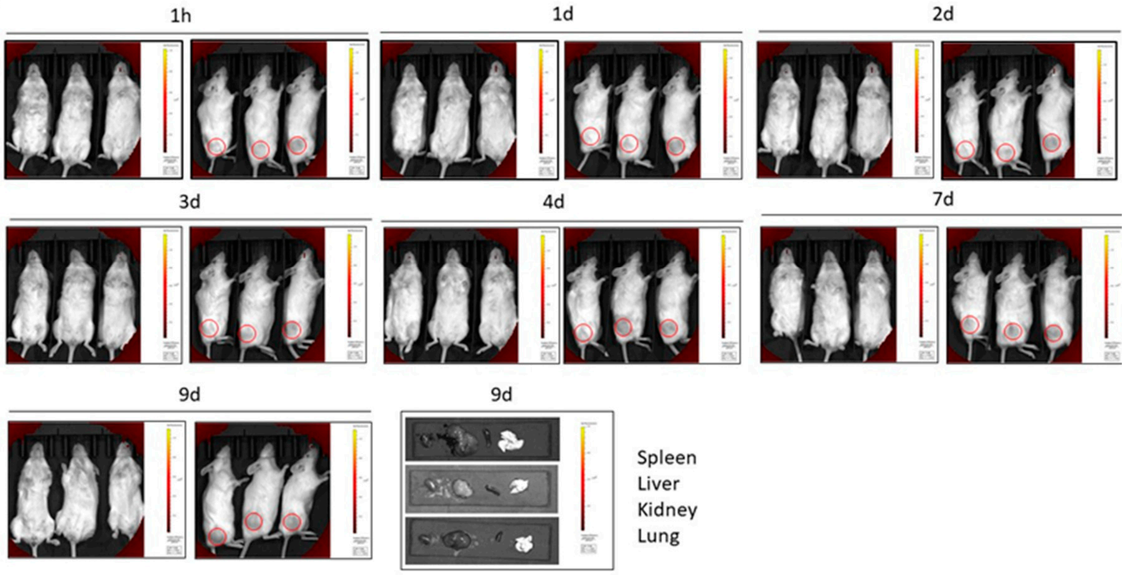

Figure S31. *In vivo* imaging of systemic biodistribution of ICG loaded nanocapsules in NSG mice. A375 challenged NSG mice were treated with vehicle (PBS) (n=3/group). Locations for tumor cells injections have been indicated with a red circle.
